# Towards a global understanding of tree mortality

**DOI:** 10.1111/nph.20407

**Published:** 2025-01-31

**Authors:** International Tree Mortality Network, Cornelius Senf, Adriane Esquivel‐Muelbert, Thomas A. M. Pugh, William R. L. Anderegg, Kristina J. Anderson‐Teixeira, Gabriel Arellano, Mirela Beloiu Schwenke, Barbara J. Bentz, Hans Juergen Boehmer, Ben Bond‐Lamberty, Kauane Maiara Bordin, Francis Q. Brearley, Filippo Bussotti, Maxime Cailleret, J. Julio Camarero, Gherardo Chirici, Flavia R. C. Costa, Ricardo Dalagnol, Hendrik Davi, Stuart J. Davies, Sylvain Delzon, Bishnu Prasad Dhakal, Renato A. Ferreira de Lima, Marco Ferretti, Joseph B. Fontaine, Matteo Garbarino, André Luís de Gasper, Arthur Gessler, Gregory S. Gilbert, John L. Godlee, Francisco Maiato Pedro Gonçalves, Leen Govaere, Alvaro G. Gutiérrez, Ernesto Gómez Cardozo, William M. Hammond, Henrik Hartmann, Martina L. Hobi, Andrés Holz, Jürgen Homeier, Mark Joseph Hovenden, Cho‐ying Huang, Bruno Hérault, Toby Jackson, Tommaso Jucker, Alistair S. Jump, Samuli Junttila, Teja Kattenborn, Joice Klipel, Martyna M. Kotowska, Kamil Král, Nicola La Porta, Leonel Lopez‐Toledo, René López‐Camacho, Eduardo Eiji Maeda, Jesús Mallol Díaz, Emanuel H. Martin, Jordi Martínez‐Vilalta, Nate McDowell, Peter W. Moonlight, Akira S. Mori, Mohd Afzanizam Muda, Jan‐Peter Mund, Robert Muscarella, Moisés Méndez‐Toribio, Sandra C. Müller, Thomas A. Nagel, Stefan Neagu, Charles Andrew Nock, Moses Nsanyi Sainge, Michael J. O'Brien, Josep Peñuelas, George L. W. Perry, Oliver L. Phillips, Juan Manuel Posada, Ricardo Ribeiro Rodrigues, Anamaria Roman, Guillaume Xavier Rousseau, Nadine Katrin Ruehr, Paloma Ruiz‐Benito, Katinka X. Ruthrof, Christian Salas‐Eljatib, Tajna Sanders, Rodrigo Scarton Bergamin, Tobias Scharnweber, Mart‐Jan Schelhaas, Bernhard Schuldt, Selina Schwarz, Rupert Seidl, Ekaterina Shorohova, Ana Carolina Silva, Geert Sioen, Jarosław Socha, Krzysztof Stereńczak, Jonas Stillhard, Dejan B. Stojanović, Susanne Suvanto, Miroslav Svoboda, Martina Sánchez‐Pinillos, Andrew J. Tanentzap, Anthony R. Taylor, Fabiano Turini Farah, Giorgio Vacchiano, Alexander C. Vibrans, Alberto Vilagrosa, Emilio Vilanova, Lars T. Waser, Susan K. Wiser, Kailiang Yu, Miguel A. Zavala, Laio Zimermann Oliveira, Daniel Zuleta, Alvaro Boson de Castro‐Faria, Ernst van der Maaten, Marieke van der Maaten‐Theunissen

**Keywords:** disturbance, forest inventory, forest monitoring, remote sensing, tree dieback

## Abstract

Rates of tree mortality are increasing globally, with implications for forests and climate. Yet, how and why these trends vary globally remain unknown. Developing a comprehensive assessment of global tree mortality will require systematically integrating data from ground‐based long‐term forest monitoring with large‐scale remote sensing. We surveyed the metadata from 466 865 forest monitoring plots across 89 countries and five continents using questionnaires and discuss the potential to use these to estimate tree mortality trends globally. Our survey shows that the area monitored has increased steadily since 1960, but we also identify many regions with limited ground‐based information on tree mortality. The integration of existing ground‐based forest inventories with remote sensing and modelling can potentially fill those gaps, but this requires development of technical solutions and agreements that enable seamless flows of information from the field to global assessments of tree mortality. A truly global monitoring effort should promote fair and equitable collaborations, transferring funding to and empowering scientists from less wealthy regions. Increasing interest in forests as a natural climate solution, the advancement of new technologies and world‐wide connectivity means that now a global monitoring system of tree mortality is not just urgently needed but also possible.

## Zusammenfassung

Die Sterblichkeitsrate von Bäumen nimmt in vielen Regionen zu, was Auswirkungen auf die Waldökosysteme und das Klima hat. Wie und warum diese Trends weltweit variieren, ist jedoch nach wie vor unbekannt. Um eine umfassende Bewertung der globalen Baumsterblichkeit zu ermöglichen, ist die systematische Verknüpfung von Daten aus bodengestütztem Langzeitmonitoring von Wäldern und großflächiger Fernerkundung sowie Modellierung notwendig. Wir haben die Metadaten von 466 865 Waldüberwachungsflächen in 89 Ländern auf fünf Kontinenten mit Hilfe von Fragebögen erhoben und erörtern das Potenzial dieser Daten, Trends in der Baummortalität zu erfassen. Unsere Erhebung zeigt, dass die überwachte Fläche seit 1960 stetig zugenommen hat. Allerdings stellen wir auch fest, dass es viele Regionen mit begrenzten bodengestützten Informationen zur Baummortalität gibt. Die Verknüpfung von bestehenden bodengestützten Waldinventuren und Waldmonitoringaktivitäten mit Fernerkundung und Modellierung kann diese Lücken schließen. Dies erfordert jedoch die Entwicklung technischer Lösungen und rechtsverbindlicher Vereinbarungen, die einen nahtlosen Informationsfluss von der Feldarbeit bis hin zu globalen Bewertungen der Baummortalität ermöglichen. Schließlich sollte ein globales Monitoring eine faire Zusammenarbeit fördern, indem es Finanzmittel an Wissenschaftler und Wissenschaftlerinnen aus weniger wohlhabenden Regionen weiterleitet und diese dadurch stärkt. Das wachsende Interesse an Wäldern und ihrem Beitrag dem Klimawandel zu begegnen, die Entwicklung neuer Technologien und die weltweite Vernetzung bedeuten, dass ein globales Überwachungssystem für die Baummortalität nicht nur dringend erforderlich, sondern auch möglich ist.

## 概括

树木死亡率在许多地区正在增加，会对森林和气候产生了影响。然而，这些全球性趋势的变化方式和原因仍不清楚。开展对全球树木死亡率的全面评估需要系统性的整合地面长期森林监测数据和大尺度遥感数据。在此，我们使用问卷调查的方式调查了来自五大洲 89 个国家的466,865个森林监测样方的元数据，并讨论了利用这些数据估算全球树木死亡率趋势的可能性。 我们的调查显示，监测面积自 1960 年以来稳步增长，但我们也发现许多地区的树木死亡率地面信息有限。将现有的地面森林资源调查和监测与遥感和建模相结合可以填补这些空白，但这需要开发技术解决方案和具有法律约束的协议，以实现从实地到全球树木死亡率评估的信息无缝流动。最后，一项真正的全球监测工作应促进公平合作，将资金转移到不太富裕地区的科学家并增强他们的技能。随着对森林作为自然气候解决方案的兴趣与日俱增、新技术的发展和全球范围内的互联互通，意味着现在全球树木死亡率监测系统的建立不仅迫在眉睫，而且也是可行的。.

## Resum

Les taxes de mortalitat dels arbres estan augmentant en moltes regions, amb importants implicacions per als boscos i el clima. Tot i així, com i per què aquestes tendències varien globalment continua sent desconegut. Desenvolupar una avaluació completa de la mortalitat global dels arbres requereix integrar sistemàticament dades del seguiment forestal a llarg termini basat en mesures de veritat‐terreny i teledetecció a gran escala. Aquí examinem les metadades de 466.865 parcel·les de seguiment forestal en 89 països i cinc continents mitjançant qüestionaris i discutim el potencial d’utilitzar aquestes dades per estimar les tendències de mortalitat dels arbres a nivell global. La nostra enquesta mostra que l’àrea monitoritzada ha augmentat constantment des de 1960, però també identifiquem moltes regions amb informació limitada sobre la mortalitat dels arbres observada sobre el terreny. La integració dels inventaris forestals i el seguiment existents sobre el terreny amb la teledetecció i la modelització pot potencialment omplir aquests buits, però això requereix el desenvolupament de solucions tècniques i acords legalment vinculants que permetin fluxos d’informació sense problemes des del camp fins a les avaluacions globals de la mortalitat dels arbres. Finalment, un esforç de monitorització realment global hauria de promoure col·laboracions justes, transferint finançament i apoderant els científics de les regions menys riques. L’augment de l’interès pels boscos com a solució natural per al clima, l’avanç de noves tecnologies i la connectivitat mundial significa que ara un sistema global de monitorització de la mortalitat dels arbres no només és urgentment necessari, sinó que és a més possible.

## Shrnutí

Míra mortality stromů se v mnoha regionech zvyšuje, což má dopady na lesy a klima. Jak a proč se tyto trendy globálně liší, však zůstává neznámé. K vytvoření komplexního hodnocení globální mortality stromů bude třeba systematicky integrovat data z dlouhodobého terénního monitoringu lesů s rozsáhlým dálkovým průzkumem Země. Zde jsme prozkoumali metadata z 466 865 monitorovacích ploch v lesích napříč 89 zeměmi a pěti kontinenty pomocí dotazníků a diskutujeme o možnosti využití těchto dat k odhadu globálních trendů mortality stromů. Naše studie ukazuje, že monitorovaná plocha se od roku 1960 neustále zvyšovala, ale zároveň identifikujeme mnoho regionů s omezenými údaji o mortalitě stromů. Integrace stávajících pozemních inventarizací a systémů monitoringu lesů s dálkovým průzkumem a modelováním může potenciálně tyto mezery vyplnit. To ale vyžaduje vývoj technických řešení a právně závazných dohod, které umožní plynulý tok informací z terénu až ke globálnímu průběžnému vyhodnocování. Skutečně globální monitorovací úsilí by nakonec mělo podporovat spravedlivou spolupráci, přenos financí a posilování postavení vědců z méně bohatých regionů. Rostoucí zájem o lesy jako o přírodní klimatické řešení, pokrok nových technologií a celosvětová propojenost znamenají, že nyní je globální monitorovací systém mortality stromů nejen naléhavě potřebný, ale také možný.

## Riepilogo

I tassi di mortalità degli alberi stanno aumentando in molte regioni del mondo, con rilevanti implicazioni ecologiche per le foreste e il clima. Tuttavia, rimangono sconosciute le dinamiche e i meccanismi attraverso i quali la tendenza all’aumento della mortalità varia a livello globale. Una comprensiva stima dei livelli delle tendenze della mortalità degli alberi richiede l’integrazione sistematica e su larga scala dei dati provenienti dal monitoraggio forestale a lungo termine tramite rilievi a terra con il telerilevamento. Nel presente lavoro vengono riassunti i dati complessivi di 466.865 aree di monitoraggio forestale distribuite in 89 paesi e cinque continenti, e viene discusso il loro potenziale per la stima delle tendenze globali della mortalità degli alberi. I dati presentati mostrano che le superfici monitorate sono aumentate costantemente dal 1960, ma identificano anche molte regioni con limitate informazioni basate sui rilievi a terra. L’integrazione degli inventari forestali esistenti (con osservazioni dirette a terra) con il telerilevamento e la modellizzazione hanno la capacità potenziale di colmare queste lacune, ma ciò richiede lo sviluppo di soluzioni tecniche e accordi giuridicamente vincolanti che consentano flussi ininterrotti di informazioni. Infine, uno sforzo per la realizzazione di un monitoraggio veramente globale dovrebbe promuovere collaborazioni eque, trasferendo finanziamenti e responsabilizzando gli scienziati delle regioni meno ricche. Il crescente interesse per le foreste come soluzione climatica naturale, il progresso delle nuove tecnologie e la connettività mondiale fanno sì che ora un sistema di monitoraggio globale della mortalità degli alberi non sia solo urgente e necessario, ma anche possibile.

## सारांश

संसारका धेरै क्षेत्रहरूमा रूखको मृत्यु दर वृद्धिका कारण वन र जलवायुमा असर परेको छ । रूखको मृत्यु दर हरुमा विविधता देखिन्छ तर विश्वव्यापी रूपमा के ले र कति मात्रामा सो विविधतामा योगदान गरेको छ भन्ने कुराको पर्याप्त लेखाजोखा हुन वाँकी छ । विश्वव्यापी रूखको मृत्यु दरको विस्तृत मूल्याङ्कन पद्धतिको विकासका लागि दीर्घकालीन अनुगमनका लागि स्थापित स्थायी नमूना प्लटहरूको तथ्याङ्कका साथै वृहत‐स्तरको दूरसम्वेदन प्रणालीमा आधारित तथ्याङ्कहरूको एकीकरणf गर्न आवश्यक छ।सो परिपेक्षमा हामीले पाँच महादेशका ८९ देशहरूबाट ४६६ ८६५ स्थायी नमूना अनुगमन प्लटहरूका बारेमा प्रश्नोत्तर विधिका माध्यमबाट तथ्याङ्कका अवयवहरूको सर्वेक्षण गरेका छौं र ती तथ्याङ्कहरूलाई प्रयोग गरेर विश्वव्यापी रूखको मृत्यु दरको प्रवृत्ति अनुमान गर्ने सम्भावनाका बारेमा छलफल गरेका छौं । यस सर्वेक्षणका अनुसार सन् १९६० देखि रूखको मृत्यु दरका सम्बन्धमा अनुगमन गरिने क्षेत्र निरन्तर रूपमा बढेको भएतापनि रूखको मृत्यु दरका बारेमा जानकारी दिने स्थायी नमूना प्लटहरू धेरै क्षेत्रमा अझै सीमित मात्रामा रहेको पाइन्छ । विद्यमान स्थायी नमूना प्लटहरूको सर्वेक्षण र अनुगमनका साथै दूरसम्वेदन तथ्याङ्क र मोडलिङ्गको एकिकरण मार्फत रूखको मुत्यु दरका बारेमा रहेको अन्योलतालाई कम गर्न सकिन्छ, तर यसका लागि समस्यालाई समाधान गर्ने प्रविधिको विकास गरी विश्वव्यापीरूपमा रूखको मृत्युदरको मूल्याङ्कन तथ्याङ्कको सहज प्रवाहलाई सक्षम पार्ने कानूनी रूपमा बाध्यकारी सम्झौताको आवश्यकता रहेको छ. अन्तमा, विश्वासिलो विश्वव्यापी अनुगमनको लागि निष्पक्ष सहकार्यलाई बढावा दिदै कम विकसित देशका वैज्ञानिकहरूलाई वित्तिय स्रोतको व्यवस्था र प्राविधिकरूपमा सशक्तिकरण गर्नुपर्छ। प्रकृतिमा आधारित जलवायु परिवर्तनको साधनको रूपमा वनलाई बढ्दो चासो, नविन प्रविधिहरूको विकास र विश्वव्यापी सहकार्यको कारण, रुखको मृत्युदरको विश्वव्यापी अनुगमन प्रणालीको विकास तत्कालको आवश्यकता मात्र नभई एक सम्भावना पनि रहेको देखिन्छ।.

## Rezumat

Rata mortalității arborilor este în creștere în multe regiuni, cu implicații asupra pădurilor și climei. Totuși, cum și de ce aceste tendințe variază la nivel global rămâne necunoscut. Dezvoltarea unei evaluări exhaustive, globale a mortalității arborilor necesită integrarea sistematică a datelor de monitorizare pe termen lung, din teren a pădurilor, cu teledetecția la scară largă. Aici analizăm metadatele din 466.865 de parcele de monitorizare a pădurilor din 89 de țări, din cinci continente, folosind chestionare și discutăm potențialul utilizării acestora pentru a estima tendințele mortalității globale ale arborilor. Deși suprafața monitorizată a crescut constant din 1960, multe regiuni au informații limitate privind mortalitatea din teren a arborilor. Integrarea inventarierilor forestiere cu teledetecția și modelarea ar putea umple aceste lacune, dar aceasta necesită dezvoltarea de soluții tehnice și acorduri legal asumate care să asigure fluxul neîntrerupt de informații din teren către evaluările globale ale mortalității arborilor. O inițiativă de monitorizare cu adevărat globală ar trebui să promoveze colaborări echitabile, direcționând finanțare și întărind capabilitățile oamenilor de știință din regiunile mai puțin bogate. Interesul în creștere față de păduri ca soluție naturală pentru climă, dezvoltarea noilor tehnologii și conectivitatea globală fac ca, în prezent, un sistem global de monitorizare a mortalității arborilor să fie nu doar urgent necesar, ci și posibil.

## Resumen

Las tasas de mortalidad de los árboles están aumentando en muchas regiones, con implicaciones para los bosques y el clima. Sin embargo, aún se desconoce cómo y por qué varían estas tendencias a escala mundial. El desarrollo de una evaluación exhaustiva de la mortalidad arbórea mundial requerirá la integración sistemática de datos de seguimiento forestal a largo plazo con teledetección a gran escala. En este trabajo se analizan los metadatos de 466.865 parcelas de seguimiento forestal en 89 países y cinco continentes mediante el uso de cuestionarios y se discute el potencial de su uso para estimar las tendencias de mortalidad arbórea a nivel mundial. Nuestro estudio muestra que el área monitorizada ha aumentado de forma constante desde 1960, pero también se han identificado muchas regiones con información limitada sobre mortalidad arbórea. La integración de inventarios y seguimientos forestales terrestres existentes junto con el uso de la teledetección y la modelización puede llenar potencialmente esas lagunas, pero para ello es necesario desarrollar soluciones técnicas y acuerdos jurídicamente vinculantes que permitan flujos de información fluidos desde el campo hasta las evaluaciones globales de la mortalidad arbórea. Por último, un esfuerzo de seguimiento verdaderamente global debería promover colaboraciones equitativas, transfiriendo financiación a los científicos de regiones menos ricas y potenciando su papel. El creciente interés por los bosques como una solución climática natural, el avance de las nuevas tecnologías y la conectividad mundial hacen que ahora no sólo sea urgente, sino también posible, un sistema mundial de seguimiento de la mortalidad de los árboles.

## Ringkasan

Kadar kematian pokok meningkat di banyak wilayah, dan memberi implikasi untuk hutan dan iklim. Namun bagaimana dan mengapa corak kematian pokok ini berbeza‐beza secara global masih tidak diketahui. Membangunkan penilaian menyeluruh tentang kematian pokok global memerlukan data secara bersepadu dan sistematik daripada pemantauan hutan jangka melalui data di lapangan dan penderiaan jauh berskala besar. Kajian ini meninjau metadata daripada 466 865 plot pemantauan hutan merentasi 89 negara dan lima benua melalui kaedah soal selidik dan membincangkan potensi untuk menganggar corak kematian pokok di seluruh dunia. Tinjauan kami menunjukkan bahawa kawasan yang dipantau telah meningkat secara berterusan sejak 1960, tetapi pada yang sama banyak kawasan yang mempunyai maklumat lapangan yang terhad berkaitan kematian pokok masih lagi belum dikenalpasti. Penyepaduan inventori hutan berasaskan tanah sedia ada dan pemantauan dengan penderiaan jauh dan pemodelan berpotensi mengisi jurang tersebut, tetapi penyelesaian teknikal dan perjanjian berasaskan undang‐undang perlu dibangunkan demi membolehkan aliran maklumat yang lancar daripada lapangan kepada penilaian global kematian pokok. Akhir sekali, usaha pemantauan secara global harus menggalakkan kerjasama yang adil dan aliran dana untuk memperkasa saintis dari kawasan kurang sumber dana. Meningkatkan minat terhadap hutan sebagai kaedah penyelesaian iklim semula jadi, memacu teknologi baharu, dan jaringan hubungan seluruh dunia bermaksud sistem pemantauan kematian pokok secara global bukan sahaja diperlukan segera, tetapi juga tidak mustahil.

## Streszczenie

Wskaźniki śmiertelności drzew rosną w wielu regionach świata, co ma wpływ na lasy i klimat. Jednak to, w jaki sposób i dlaczego trendy te różnią się w skali globalnej, pozostaje nieznane. Opracowanie kompleksowej oceny globalnej śmiertelności drzew będzie wymagało systematycznej integracji danych z naziemnego długoterminowego monitorowania lasów oraz wykorzystania teledetekcji w dużej skali przestrzennej. W niniejszym artykule przeprowadziliśmy badanie metadanych z 466 865 powierzchni monitorujących lasy w 89 krajach i na pięciu kontynentach za pomocą kwestionariuszy i omówiliśmy możliwość ich wykorzystania do oszacowania trendów śmiertelności drzew na świecie. Nasze badania pokazują, że globalnie od 1960 roku monitorowany obszar lasów stale rośnie, ale identyfikujemy również wiele regionów z ograniczonymi informacjami naziemnymi na temat śmiertelności drzew. Integracja istniejących naziemnych inwentaryzacji i monitorowania lasów z teledetekcją i modelowaniem może potencjalnie wypełnić te luki, ale wymaga to opracowania rozwiązań technicznych i prawnie wiążących umów, które umożliwią płynny przepływ informacji zbieranych w terenie do globalnych ocen śmiertelności drzew. Wreszcie, prawdziwie globalny wysiłek w zakresie monitorowania powinien promować uczciwą współpracę, przekazując fundusze i wzmacniając pozycję naukowców z mniej zamożnych regionów. Rosnące zainteresowanie lasami jako naturalnym rozwiązaniem klimatycznym, rozwój nowych technologii i ogólnoświatowa łączność oznaczają, że obecnie globalny system monitorowania śmiertelności drzew jest nie tylko pilnie potrzebny, ale także możliwy.

## Краткое содержание

Во многих регионах отмечено значимое для системы «лес и климат» увеличение смертности деревьев. В глобальном аспекте тренды и причинно‐следственные связи, определяющие скорость отпада деревьев, неизвестны. Разработка системы всесторонней глобальной оценки древесного отпада требует систематической интеграции данных наземного мониторинга и дистанционного зондирования. Мы анализируем возможность использования метаданных 466 865 пробных площадей, заложенных в 89 странах на пяти континентах, полученных в результате опроса специалистов для оценки глобальной скорости отпада деревьев. Согласно нашим исследованиям, площадь мониторинга постоянно увеличивается, начиная с 1960 года. Однако во многих регионах информации о скорости древесного отпада недостаточно. Пробелы в данных могут быть частично восполнены с помощью материалов дистанционного зондирования и моделирования, что, в свою очередь, требует новых технических решений и юридически обязательных соглашений, обеспечивающих глобальные потоки информации о скорости древесного отпада. В конечном счете, глобальная инициатива сотрудничества должна обеспечить перевод финансирования и расширение возможности ученых из менее экономически развитых регионов. Растущий интерес к лесам, как к природному климатическому решению, развитие новых технологий и глобальная конвергенция обусловливают не только срочную востребованность, но и возможность развития глобальной системы мониторинга скорости динамики отпада деревьев.

## Резиме

Stope mortaliteta drveća rastu u mnogim regionima, sa implikacijama na šume i klimu. Ipak, kako i zašto ovi trendovi variraju na globalnom nivou, ostaje nepoznato. Razvijanje sveobuhvatne procene globalnog mortaliteta drveća zahtevaće sistematsku integraciju podataka dugoročnih praćenja stanja šuma sa zemlje i podataka daljinske detekcije. Ovo istraživanje obuhvata meta podatke sa 466.865 parcela monitoringa šuma u 89 zemalja i pet kontinenata koristeći upitnike, diskutujući o potencijalu da se oni koriste za procenu globalnih trendova mortaliteta drveća. Naše istraživanje pokazuje da su se osmatrana i istraživane oblast stalno povećavala od 1960. godine do danas, ali takođe identifikuje mnoge regioni sa ograničenim informacijama o sušenju šuma. Integracija postojećih inventura šuma i monitoringa sa daljinskim detekcijom i modeliranjem potencijalno može popuniti te praznine, ali to zahteva razvoj tehničkih rešenja i pravno obavezujućih sporazuma koji bi omogućili nesmetan protok informacija sa terena sve do globalnih procena mortaliteta drveća. Konačno, istinski globalni napori za praćenje trebalo bi da promovišu poštenu saradnju, prebacujući sredstva i osnažujući naučnike iz manje bogatih regiona. Sve veće interesovanje za šume kao prirodno klimatsko rešenje, unapređenje novih tehnologija i svetsko povezivanje znači da sada globalni sistem praćenja smrtnosti drveća nije samo hitno potreban, već́ i moguć́.

## Yhteenveto

Puiden kuolleisuus kasvaa monilla alueilla, mikä vaikuttaa metsiin ja ilmastoon. Silti, emme edelleenkään tiedä kuinka ja miksi nämä trendit vaihtelevat globaalisti. Kattavan maailmanlaajuisen järjestelmän kehittäminen puiden kuolleisuuden arviointiin vaatii systemaattisia ja pitkäaikaisia maastokoealoja ja niiden yhdistämistä laajamittaiseen kaukokartoitukseen. Tässä tutkimme metatietoja 466 865 metsien seurantakoealasta 89 maassa viidellä mantereella kyselylomakkeiden avulla ja pohdimme niiden käyttämisen mahdollisuuksia puiden kuolleisuustrendien arvioimiseksi globaalisti. Tutkimuksemme osoittaa, että seurattu pinta‐ala on kasvanut tasaisesti vuodesta 1960, mutta tunnistamme myös monia maantieteellisiä alueita, joilla on rajoitetusti maastoon perustuvaa tietoa puiden kuolleisuudesta. Nykyisten maastoon perustuvien metsäinventointien ja ‐seurantojen yhdistäminen kaukokartoitukseen ja mallinnukseen voi mahdollisesti täyttää nämä aukot, mutta tämä vaatii teknisten ratkaisujen kehittämistä ja oikeudellisesti sitovia sopimuksia, jotka mahdollistavat saumattoman tiedonkulun kentältä globaaleihin puiden kuolleisuuden arviointeihin. Lopuksi, todellisen globaalin seurantajärjestelmän tulisi edistää reiluja yhteistyömuotoja ja rahoituksen siirtämistä vähemmän varakkaiden alueiden tutkijoille heidän voimaannuttamiseksi. Kasvava kiinnostus metsiin luonnollisena ilmastoratkaisuna, uusien teknologioiden kehitys ja maailmanlaajuinen yhteydenpito tarkoittavat, että nyt globaali puiden kuolleisuuden seurantajärjestelmä ei ole vain kiireellisesti tarpeen, vaan myös mahdollinen.

## Résumé

La mortalité des arbres ne cesse d’augmenter dans de nombreuses régions du monde, ce qui a des répercussions sur les forêts et le climat. Cependant, nous ne connaissons pas les causes et la variabilité de ces tendances à l’échelle mondiale. L’évaluation complète de la mortalité des arbres à l’échelle de la planète nécessite l’intégration systématique des données issues de la surveillance à long terme des forêts sur le terrain et de la télédétection à grande échelle. Nous avons étudié ici les métadonnées des inventaires forestiers dans 89 pays et sur cinq continents à l’aide de questionnaires et nous discutons de la possibilité de les utiliser pour estimer les tendances de mortalité à l’échelle mondiale. Notre étude montre d’une part que la superficie forestière inventoriée a régulièrement augmenté depuis les années 60, et d’autre part qu’il existe de nombreuses régions pour lesquelles les informations au sol sur la mortalité des arbres sont limitées. L’intégration des inventaires forestiers, de la surveillance par télédétection et de la modélisation peut potentiellement combler ces lacunes, mais cela nécessite le développement de solutions techniques et d’accords juridiquement contraignants pour permettre un flux continu d’informations depuis le terrain jusqu’aux évaluations globales. Enfin, un véritable effort mondial de surveillance devrait promouvoir des collaborations équitables, en transférant des fonds aux scientifiques des régions les moins riches et en leur donnant les moyens d’agir. Dans le contexte du changement climatique, l’intérêt croissant pour les forêts en tant que solutions fondées sur la nature, les progrès des nouvelles technologies et la connectivité mondiale signifient qu’aujourd’hui un système global de surveillance de la mortalité n’est pas seulement une nécessité urgente, mais aussi une action réalisable dès à présent.

## まとめ

世界中の多くの地域で樹木の枯死率は増加しており、森林や気候に影響を及ぼしつつある。しかしながら、こうした傾向が世界的にどのように変化しているのか、また、なぜ変化しているのかは、依然として不明である。世界の樹木死亡率を包括的に評価するには、地上ベースの長期森林モニタリングと大規模リモートセンシングのデータを系統的に統合する必要がある。本研究では、5大陸89カ国にまたがる466,865カ所の森林モニタリングプロットのメタデータをもとに質問票を用いた調査研究を実施し、世界全体の樹木死亡率の傾向を推定した。その結果、1960年以降、モニタリング対象面積は着実に増加している一方で、樹木の死亡率に関する実際のグラウンドベースの情報が限られている地域も依然と多いことが確認された。既存の森林インベントリやモニタリングとリモートセンシングやモデリングを統合することで、これらの知識ギャップを埋められる可能性があるが、そのためには、現場から樹木死亡率のグローバルな評価へのシームレスな情報パイプラインを可能にする技術開発、政策的枠組の担保が必要である。最後に、本当の意味での地球規模モニタリングの取り組みは、、資金の不足している地域の研究者への支援強化、さらなる能力開発、公平かつ衡平な協力関係を促進がなされるべきである。自然に根差した解決策に基づく気候変動対策としての森林への関心の高まり、新技術の進歩、そして世界的なつながりは、森林の変化、樹木死亡率の変化を探知するための世界的モニタリングシステムが緊急に必要とされているだけでなく、実装可能であることを意味している。.

## Resumo

As taxas de mortalidade de árvores estão aumentando em muitas regiões, com implicações para as florestas e o clima. No entanto, como e por que essas tendências variam globalmente ainda é desconhecido. Desenvolver uma avaliação abrangente da mortalidade global de árvores exigirá a integração sistemática de dados de monitoramento florestal de longo prazo em solo com sensoriamento remoto em grande escala. Neste estudo, avaliamos metadados de 466.865 parcelas de monitoramento florestal em 89 países e cinco continentes com o uso de questionários e discutimos o potencial de usá‐los para estimar tendências de mortalidade de árvores globalmente. Nossa pesquisa demonstrou que as áreas monitoradas aumentaram de forma constante desde 1960, mas também identificamos muitas regiões com informações limitadas sobre a mortalidade de árvores quando essas informações são baseadas em solo. A integração dos inventários florestais e do monitoramento existentes, baseados em solo, com sensoriamento remoto e modelagem pode potencialmente preencher essas lacunas, mas isso requer o desenvolvimento de soluções técnicas e acordos juridicamente vinculativos que permitam fluxos contínuos de informações do campo para avaliações globais de mortalidade de árvores. Finalmente, um esforço de monitoramento verdadeiramente global deve promover colaborações justas, transferindo financiamento e capacitando cientistas de regiões menos ricas. O crescente interesse nas florestas como uma solução climática natural, o avanço de novas tecnologias e a conectividade mundial significam que agora um sistema global de monitoramento da mortalidade de árvores não é apenas urgentemente necessário, mas também possível.

## Introduction

Increases in tree mortality over time have been detected in forest ecosystems around the globe (Brienen *et al*., 2015; McDowell *et al*., 2018, 2020; Senf *et al*., 2021; Hammond *et al*., 2022). The reported increases in tree mortality have been associated with anthropogenic climate change via increasing climate extremes, such as heat (Breshears *et al*., 2009), atmospheric aridity (Allen *et al*., 2015; Grossiord *et al*., 2020), soil drought (Allen *et al*., 2010; Senf *et al*., 2020), fire severity (Abatzoglou & Williams, 2016; Ward *et al*., 2020; van Wees *et al*., 2021), storms (Uriarte *et al*., 2019; Senf & Seidl, 2021b), insect outbreaks (Kurz *et al*., 2008; Weed *et al*., 2013; Seidl *et al*., 2017), and spread of invasive insects and pathogens (Anderson‐Teixeira *et al*., 2021). Widespread increases in tree mortality will have pervasive and long‐term impacts on global forest ecosystems, their biodiversity and the ecosystem services they provide (Hartmann *et al*., 2018b; McDowell *et al*., 2020).

Understanding trends in, and causes of, tree mortality globally is crucial for climate change mitigation efforts, because forests have for decades been responsible for a net annual uptake of *c*. 20% of the carbon dioxide released by human activities (Pan *et al*., 2011; Pugh *et al*., 2019; Harris *et al*., 2021). Yet, projections of the future of this sink diverge dramatically, with tree mortality rates emerging as one of the key uncertainties (Friend *et al*., 2014; Wu *et al*., 2018; Hubau *et al*., 2020; Pugh *et al*., 2020). With only very tight carbon budgets of *c*. 100 Pg C remaining to hold global temperatures within 1.5° of preindustrial levels (Friedlingstein *et al*., 2022), changes in forest regions can have substantial implications for national commitments required to reach this temperature target. For instance, the 2010 Amazon drought is estimated to have led to a regional reduction in carbon uptake of 0.5 Pg C (Potter *et al*., 2011). Uncertainties in tree mortality rates also hang over the long‐term efficacy of restoration programmes, widely touted as a key natural climate solution (Cook‐Patton *et al*., 2021). But forests are of interest for much more than climate change mitigation services. Understanding tree mortality trends is also fundamental to developing policies that can effectively support or enhance biodiversity, as it is for developing management plans that effectively deliver required wood supplies. Reducing the uncertainties in forest futures requires substantial increases in the accuracy of tree mortality representations in modelling tools. Understanding the present is a prerequisite to building robust predictions about the future, and regions being affected by increased mortality today can provide early warnings for their neighbours. Currently, however, monitoring of tree mortality globally is fragmented and inconsistent. Scientists and society thus lack a clear, accurate, and consistent assessment of rates and trends of tree mortality across the globe. This urgently needs to be resolved.

**Fig. 1:**
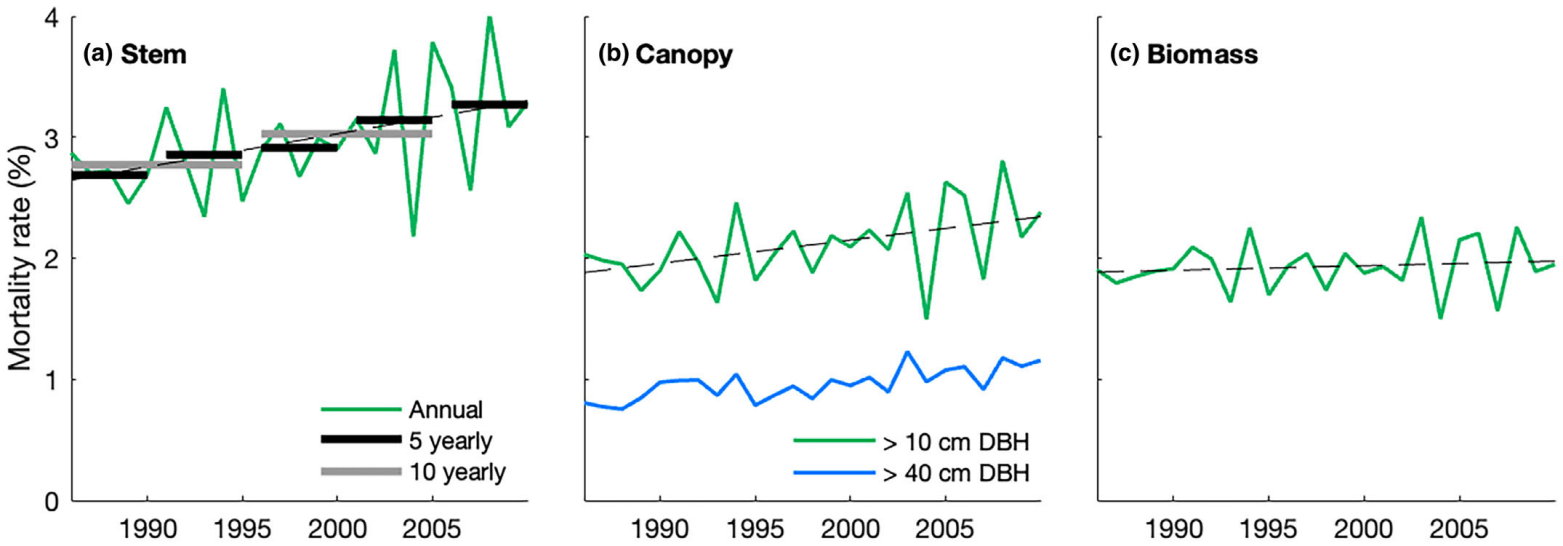
Different indicators and aggregations of mortality rates can give very different impressions of the dynamics they describe. (a) Stem mortality rates can vary substantially from year to year, information which is lost in the 5‐ or 10‐yr aggregations typically used in ground‐based inventories. Shown here for stems > 10 cm diameter at breast height (DBH). (b) Mortality rates based on canopy area, as typically assessed from satellites, can differ substantially from stem mortality rates. When only trees large enough to be picked up by long‐running satellite sensors like Landsat are considered (> 40 cm DBH; Scheel *et al*., 2022), such differences can be even larger (blue line). (c) Biomass mortality rates are dominated by big trees, whilst stem mortality rates are dominated by small trees (Piponiot *et al*., 2022). This means that trends can differ dramatically between the different metrics. The linear trend for trees > 10 cm DBH for stem, canopy and biomass mortality rate (dashed line) is 0.03, 0.02 and 0.00% yr^−1^, respectively. Example rates shown here are self‐consistent and calculated based on simulations with the LPJ‐GUESS vegetation model for forests in Central Europe by Scheel *et al*. (2022).

Monitoring changes in tree mortality is a challenging task. For over a century, foresters, scientists and government bodies have been monitoring forests by ground‐based surveys of attributes, such as tree size, species identity, crown condition and whether trees are alive (Breidenbach *et al*., 2020). Yet, traditional forest surveys were rarely designed specifically to monitor mortality: with few exceptions, they have long remeasurement intervals (typically > 4 yr) (Ståhl *et al*., 2012), which – combined with the stochastic nature of tree mortality – makes tracking changes in tree mortality over time and attribution of causality difficult (Fig. 1). Furthermore, many forested regions lack standardised forest monitoring systems that assess the fate of individual trees due to logistical, financial, social or political reasons. Novel technologies from remote sensing can add insight over large scales, but challenges remain in monitoring the internal dynamics, such as changes in forest structure, composition or mortality, as well as in relating the observed changes to ground‐based monitoring (Fig. 1). Bringing together diverse efforts and protocols across platforms, alongside filling geographical gaps in monitoring efforts, remains a large, yet resolvable, challenge (Zweifel *et al*., 2023).

Here, we provide a framework to systematically and continuously monitor trends in tree mortality by synthesising existing data, analogous to concepts adopted by the climate science community (Harris *et al*., 2020), providing information to inform national, regional and global policy. Specifically, we: (1) define the minimum requirements of ground‐based forest monitoring data to identify trends in tree mortality; (2) review existing ground‐based monitoring networks covering 89 countries across all forested continents; (3) discuss ways to close data gaps and improve data integration; and (4) highlight approaches to promote fair collaborations to overcome the underrepresentation of scientific knowledge from particular regions. Our framework provides a base to generate long‐term monitoring of trends in tree mortality and to make robust predictions about future changes in tree mortality globally.

## Minimum data requirements to capture trends in tree mortality

**Fig. 2:**
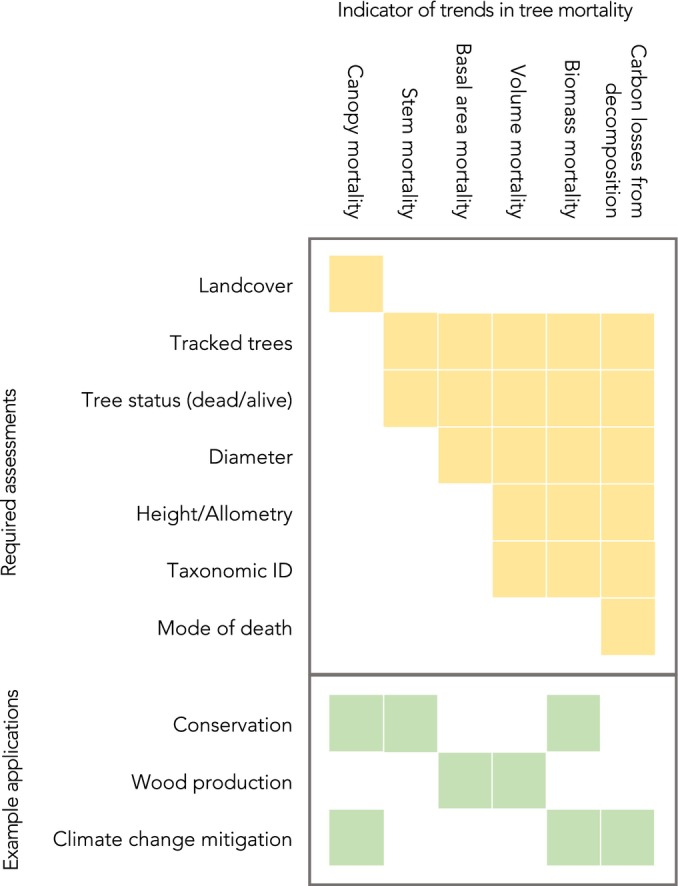
Minimum requirements for assessing different indicators of trends in tree mortality and applications of each indicator. Coloured squares represent the requirement of these measurements for the trend of interest. Decomposition is included because of its carbon cycle implications, where mode of death (e.g. standing, fallen and harvested) is key.

Quantifying trends in forest dynamics, including tree mortality, requires linking repeated observations in time and space. At coarse scales, trends in canopy openings are now available from continental and global‐scale satellite products (Hansen *et al*., 2013; White *et al*., 2017; Senf & Seidl, 2021a). These products provide an overview of areas of temporary tree cover loss due to large disturbance events. Whilst valuable, they do not resolve individual trees and lack information on sub‐canopy tree mortality and thus provide only limited, and indirect, insights into how increasing tree mortality is affecting wood production, conservation or climate change mitigation efforts. At finer scales, stem mortality rates measured from assessments of tree status in cyclic forest inventories give an indication of the probability of survival of individuals of a given tree species at a given location (Esquivel‐Muelbert *et al*., 2019). Combining this status information with tree size and allometric relationships enables the calculation of basal area or wood volume loss rates, which are key indicators for monitoring tree mortality in forestry (Yu *et al*., 2019). Biomass and carbon losses can be calculated in a similar manner and are vital to understanding whether the carbon sink in the forest is changing (Hubau *et al*., 2020). Each of these indicators provides key parameters for different areas of science and policymaking and has different minimum required measurements (Fig. 2).

Beyond quantifying trends to understanding causes and drivers, it is also crucial to inform those responsible for managing forests, yet this presents a range of challenges. For instance, attributing observed tree mortality to specific disturbance events requires frequent observations (e.g. annual or even intra‐annual compared with the 5–10 yr typical of National Forest Inventories (NFIs)) or retrospective approaches, such as tree‐ring data (Schurman *et al*., 2018). Detailed information on local environmental conditions, such as topography and soil type, is also crucial for understanding causal relationships between tree mortality and environmental changes (Brun *et al*., 2020; Stereńczak *et al*., 2020; Costa *et al*., 2023). Assessing the relative fitness of different tree species or functional strategies, key for informing climate change adaptation efforts, requires species‐ (and/or trait) specific data. Monitoring of defoliation, insect and pathogen occurrence and management actions provide insights into drivers of trends, as does association with high‐quality meteorological observations. All this information needs to be brought together at a spatial scale that is fine enough to have tree‐scale relevance, but coarse enough to allow assessments at a global scale. An idealised monitoring scheme that can both assess trends in tree mortality and facilitate attribution of causes and drivers must comprise:

1. A continuous time series with at least 5‐yearly resolution of status of individual trees (alive/dead) paired with more frequent complementary observations at annual resolution. Annual resolution allows to link mortality to climatic events with much greater certainty than 5–10‐yr intervals, greatly improving attribution. It also improves the quality of assessments in point number 5 below and crucially allows timely identification of changes in mortality rates.
2. Representativeness across both geographical and environmental gradients (e.g. topography) to enable characterisation at the landscape scale and up.
3. Identification of species and structural characteristics (diameter and biomass) of surviving and dead individuals. This information is crucial to calculate indicators beyond stem mortality (point number 4) and to diagnose which types and sizes of trees are most being affected.
4. Multiple indicators of mortality to support different applications (Fig. 2).
5. Information on the mode of death. At its most basic level, this should cover whether a tree died standing, broken, uprooted or was harvested. This information in combination with assessment of the presence of charcoal may allow for the attribution of potential causes of death, such as droughts, fires and storms.
6. Standardising the above points 1 through 5 across the globe and making the observations rapidly accessible to scientists and the wider public.

Whilst annual field surveys clearly bring benefits in terms of attributing mortality to drivers (Das *et al*., 2016; Arellano *et al*., 2021), the labour‐intensiveness of such surveys makes them unpractical at scale in the real world. We assess that a 5‐yr time resolution is not unrealistically intensive, being already applied in many national surveys (Fridman *et al*., 2014; Talarczyk, 2014); but it allows for reasonably timely identification of death. Complementary approaches to provide annual information paired to the full assessment include: (1) annual mortality and disturbance agents assessments on a subset of plots, for example as applied by ICP Forests across Europe (Ferretti, 2013), or targeting a subset of trees, as applied by ForestGEO (Arellano *et al*., 2021); (2) remote sensing assessments of the individual plots, possibly linked to targeted sampling following periods of stress; or (3) scheduling of re‐censuses such that 20% of plots, broadly distributed across the monitored region, are revisited each year (Fridman *et al*., 2014; Talarczyk, 2014). An effective global dissemination system for results, such as that now available for deforestation (www.globalforestwatch.org), is also required, such that the latest knowledge from science can quickly be disseminated to society and to inform national and international policy decision‐makers governing the future of the world's forests.

## Currently available global ground‐based monitoring

A comprehensive assessment of the current state of long‐term forest monitoring data is the first step towards contextualising the currently available global understanding of trends in tree mortality. This includes assessing the potential, and limitations, of existing *in situ* forest monitoring initiatives to quantify changes in tree mortality over time. To achieve this goal, we conducted an online survey among foresters and researchers, distributed through the International Tree Mortality Network (https://www.tree‐mortality.net) and social media. The survey provided us with methodological information and metadata on where and how tree mortality has been monitored across the globe. We also actively searched for plot networks and approached people individually to respond to the questionnaire, as well as adding information on NFIs where they are available online.

**Fig. 3:**
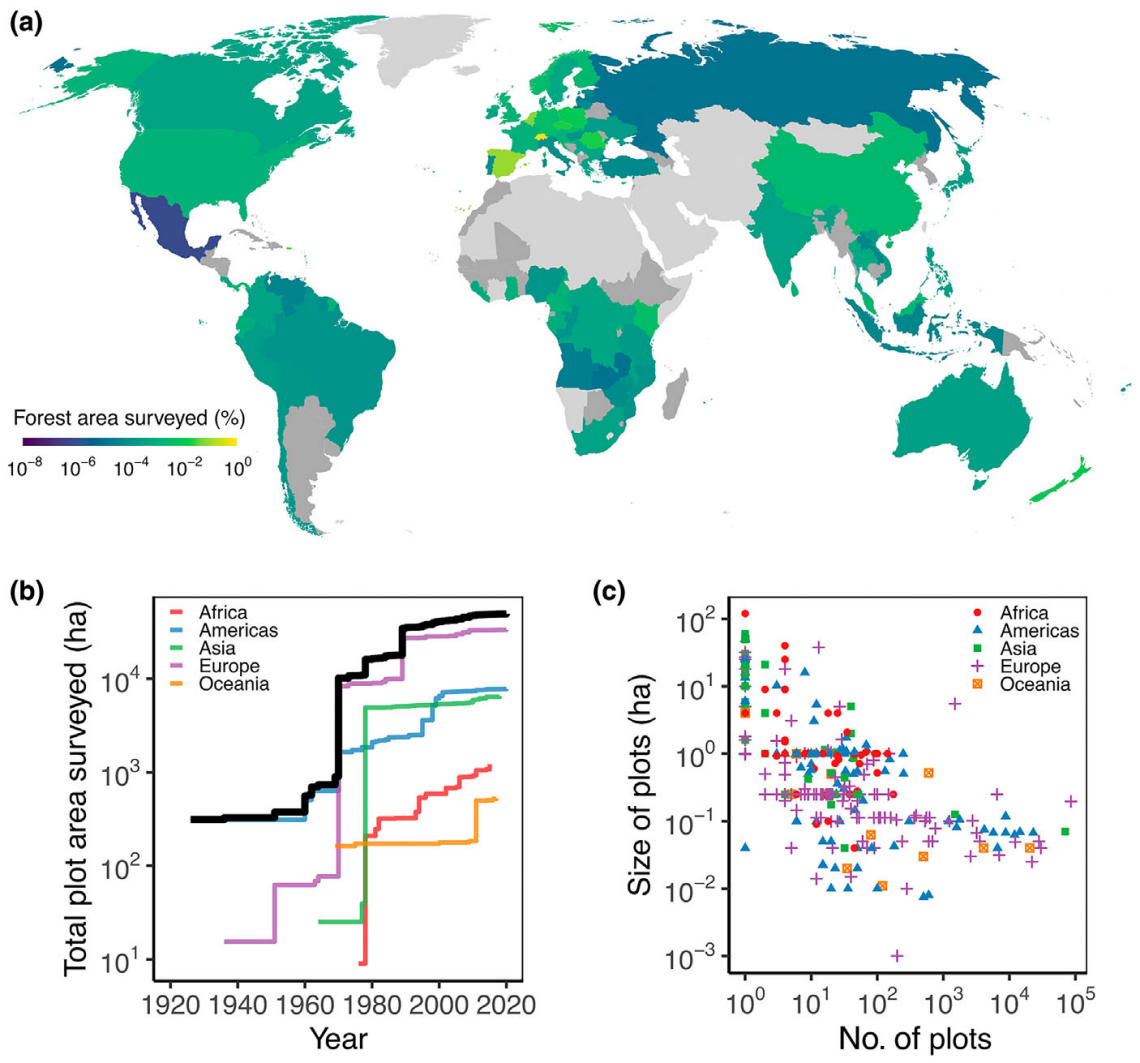
Summary of the spatial and temporal coverage of forest plots according to our survey of global mortality monitoring plots: (a) percent of the total forest area surveyed per country, (b) plot area surveyed over time for different continents and globally (black line), and (c) the distribution of size and number of plots. For countries coloured dark grey in (a), we did not receive any responses in our survey. Countries coloured light grey are countries with < 10% of their terrestrial land area covered by forests.

After filtering for duplicates and entries with missing information on the number of plots or plot size, our survey had a total of 316 entries from 89 countries on all forested continents. The total number of sampling sites was 466 865, and the total sampling site area covered by all monitoring networks together was > 40 500 ha (*c*. 0.001% of the global forest area), but sampling area varied greatly among countries and over time (Fig. 3). The forest area monitored grew rapidly and steadily after 1960 (Fig. 2b). Russia, West Africa and Central America stand out as having the poorest coverage in terms of area surveyed (Fig. 3a). Sampling in Africa was further concentrated in only relatively few large plots, with poorer areal coverage (Fig. 3c). However, we note that these lower‐recorded areas may also reflect less fluid communications with researchers in those countries. In general, there was a negative relationship between plot size and number of plots, with countries having few plots tending to have larger (research) plots, whereas countries with many plots tend to have smaller plots (likely inventory plots).

From those monitoring initiatives where metadata was available (36%, 114 out of 316 entries), the overwhelming majority track trees (94%) and all of those except one record tree status at every census (dead/alive), thus fulfilling the minimum requirement to calculate stem‐based mortality (see Fig. 2). Two‐thirds of the entries that track trees (66%) record plots at least every 5 yr and 86% revisit the plots at least every 10 yr, but only 11% of plots are annually surveyed as required to support a process‐based understanding of tree mortality. Filtering for initiatives that track trees and tree status at least every 5 yr we found that this requirement was present in only 62% of the monitoring initiatives (71 out of 114 where full information was available). Except for two cases, all initiatives tracking trees also record tree diameter, thus allowing for estimation of (at least) basal area mortality (Fig. 2). Some of the forest monitoring initiatives in our survey also collect information on tree condition (e.g. defoliation and discoloration; 38%), the potential causes of death (e.g. biotic, fire and wind throw; 68%) or whether the plot is subject to harvest (64%).

## Improving monitoring of tree mortality – a perspective

### How to fill ground‐based monitoring gaps?

There are several potential routes to narrow the existing gaps in ground‐based monitoring on spatial and temporal levels, as well as the types of indicators available (Fig. 1). National monitoring networks, such as NFIs are often relatively well funded for the long term. Protocols for these networks have typically been developed for capturing timber stocks, rather than assessing mortality rates. Ensuring that a subset of plots track individual trees across repeated censuses would fill several monitoring gaps. Decreasing the interval between NFIs from *c*. 5–10 yr to 1–2 yr would be ideal, but likely prohibitively expensive for most agencies. A realistic approach could, for instance, include a few frequently monitored but spatially representative plots (Ferretti, 2013), rapid censuses, which focus only on assessing mortality (Arellano *et al*., 2021) (potentially including standardised protocols to identify major proximate mortality causes; Das *et al*., 2016) or rolling assessment designs with a representative subset of plots being recensused each year. Furthermore, development of low‐effort protocol modifications to collect targeted ancillary variables can help to attribute drivers/causes of mortality. For instance, assessing additional plot‐level variables, such as signs of management or biotic damage, would be quicker than collecting detailed tree‐level variables, whilst still providing important information on trends in forest condition (Hartmann *et al*., 2018b). Such methods are already well documented in protocols employed by some agencies (Pollard, 2006).

In regions without regular national forest assessment programmes, our survey often identified substantial numbers of research plots. Developing cooperation between researchers who manage these plots, who often study distinct topics, can provide powerful information, even if their research does not address systematic sampling in space. Initiatives, such as ForestPlots.net (ForestPlots.net *et al*., 2021), the Tropical managed Forests Observatory (TmFO) (Sist *et al*., 2015) and ForestGEO (Anderson‐Teixeira *et al*., 2015) that connect researchers to facilitate standardising protocols and metadata and to curate data, provide examples of how to fill major data gaps in mortality trends (Hubau *et al*., 2020). Many such plots exist with one or two censuses as a basis for mortality estimations. Where plot locations have been recorded accurately, prioritising remeasurement of these plots, with protocols appropriate for capturing mortality, could dramatically increase the area under observation for mortality trends. In some regions, new plots must be established. The costs of establishing new intensive monitoring campaigns could be offset by integration with remote sensing or targeted sampling approaches to reduce the required intensity of ground sampling, particularly in tropical regions where fieldwork is more challenging (see the ‘Bringing it all together – data integration across scales’ section). Even where new NFIs are currently being established, research plots remain crucial because of their long‐term record, often stretching back decades (Phillips & Gentry, 1994; ForestPlots.net *et al*., 2021), which provide necessary context for the rates that are observed in the present day. However, relying on these research plots raises questions of research equity and the fair share of research rewards (see the ‘A comprehensive and fair global network’ section).

### The role of remote sensing

Remote sensing is often seen as a promising tool for filling gaps in monitoring tree mortality (Hartmann *et al*., 2018a). This applies especially to satellite remote sensing systems, which can provide consistent and spatially explicit information on land cover (including trees) from anywhere around the world. Yet, there are often misconceptions around what most remote sensing systems actually measure with respect to tree mortality. First and foremost, most satellite remote sensing systems provide a bird's‐eye view on trees. That is, they only give information on changes in canopy trees and – with some exceptions explained below – not on the full cohort of trees within a stand. Second, most satellite remote sensing systems record changes in spectral reflectance over time. Whilst this can serve as a proxy for tree vitality (Buras *et al*., 2020), they do not directly measure tree mortality, and models translating the changes in spectral reflectance properties into measures of tree mortality are needed. However, those models require proper calibration and validation (Senf *et al*., 2017; Cotrozzi, 2022). Third, most satellite remote sensing systems provide an aggregated signal at a spatial grain coarser than individual trees (typically 10–100 m), which makes it challenging to relate the state of individual trees to the signal recorded by the sensor. Due to those limitations, trends derived from satellite remote sensing represent total canopy cover loss rather than trends in mortality of individual trees (Fig. 1). This can challenge communication between remote sensing scientists and users of remote sensing products. Nevertheless, thanks to the outstandingly long and free‐to‐access archives of national space agencies (Wulder *et al*., 2022), mapping trends in tree cover loss is operational globally (Hansen *et al*., 2013). Many ongoing changes in forest, such as increased natural disturbances or illegal logging, would thus remain undetected without the broad‐scale view provided by satellite remote sensing. This applies especially to spatial patterns of tree canopy change (e.g. patch‐size distributions; Jucker, 2022), which cannot be characterised well with plot‐based inventories.

Novel remote sensing data and technologies enable increasingly detailed analyses that might become operational in the future. For example, commercial satellite data providers operate a series of satellites with passive optical sensors of high (< 5 m) and very high (< 1 m) spatial resolution, which have potential for detecting individual tree loss (Guo *et al*., 2007; Meddens *et al*., 2011; Brodrick & Asner, 2017). The most prominent example of this kind of data comes from the Planet missions, from which mosaics for the entire tropics were recently made available through Norway's International Climate and Forest Initiative (https://www.nicfi.no). Yet, despite a finer spatial resolution, those systems suffer from the same drawbacks as coarser sensor systems relying on reflectance in the optical wavelength region: they only provide information on trees in the forest canopy and models are required to map spectral changes to actual tree mortality. Remote sensing systems and technologies like Light Detection And Ranging (LiDAR) can overcome some of those challenges, enabling assessment of changes in canopy structure following tree death directly (Dalagnol *et al*., 2021; Cushman *et al*., 2022; Huertas *et al*., 2022). However, repeated LiDAR surveys are costly and limited in their spatial extent due to the need for aircrafts or uncrewed aerial vehicles (UAVs). An operational global monitoring of tree mortality at the individual tree or biomass level would thus require major investment into data acquisition (e.g. global repeated aerial LiDAR campaigns), which might be complemented by novel spaceborne systems, such as from the Global Ecosystem Dynamics Investigation (GEDI) mission.

### Bringing it all together – data integration across scales

The development of a monitoring system for tree mortality will depend on whether we can successfully integrate the existing wealth of data from different sources and scales, both temporal and spatial. This includes harmonising different sources of ground data, and integrating ground and remote sensing data. Process‐based forest models may help this integration take place.

#### A consistent meta‐network

Sampling designs and field measurement protocols for monitoring tree mortality differ among networks and monitoring programmes (e.g. ForestGEO and national inventories), for example in plot size, recensus frequency, sampling density across the landscape and classifications of mortality cause. Such differences emerge from the diversity of focal research questions or applications (e.g. description of stand composition vs dynamics). Whilst fully standardising designs and protocols across all networks is unrealistic, and probably also undesirable because of the different motivations underlying surveys, much could be done to reduce unnecessary differences, for example in definitions and classifications. This would greatly reduce the challenges in comparing information from different networks. Intergovernmental organisations like Forest Europe and the European Forest Institute, which deliver advice to forest ministries across many European countries, or international forest monitoring (e.g. the UN ECE ICP Forests) and forestry steward organisations like FAO or IUFRO, may foster such initiatives for harmonising protocols and even sampling designs.

Yet, much can also be done to improve harmonisation of data *post hoc*. Such harmonisation spans from what definition of forest is used as a basis for aggregation, through to diameter thresholds for sampling and the allometric equations applied. Given an appreciation of the differences in protocols, commonalities of data from different sources should be identified and, if true conformity across datasets is not possible, crosswalks should be established by looking for the ‘lowest common denominator’. Achieving this will require empirical studies that evaluate comparability of data collected by different protocols. Overall, the size of the task to harmonise data will depend on the application, differing, for instance, if the aim is to understand implications of tree mortality for stand‐level biomass or to compare mortality rates between different species or functional groups. Key to facilitating all these efforts is reporting of adequate metadata of sampling designs, field protocols and the data workflows used to create aggregated products.

#### Efficient gap filling

Remote sensing can help in filling spatial gaps in tree mortality monitoring and/or to increase the temporal density of existing inventories. To make remote sensing truly useful for filling gaps in ground‐based monitoring of tree mortality, there needs to be improved integration of remote sensing and field‐based data. This will facilitate both remote sensing model calibration/validation and the complementing of field‐based measurements with the high temporal frequency and spatial view of remote sensing data. However, integrating remote sensing and field data is challenging. Issues arise from, among others, difficulties in matching plots to pixels due to missing spatial coordinates or low geolocation accuracies (e.g. many NFIs will only provide approximate coordinates due to data privacy issues or low accuracy georeferencing), complex terrain (area seen from space differs from area on the ground), often much smaller plot than pixel sizes, missing information on whether the tree occupies the canopy or is confined to the understory (i.e. whether the tree will be exposed to air‐ and spaceborne remote sensing), or a temporal mismatch between field and remote sensing data acquisition (especially for historical data). There thus is a need for adapting field protocols to allow better integration of field and remote sensing data. Whilst changing some aspects of field protocols is difficult without losing backwards compatibility, minor adjustments will cause large improvements, for example exactly defining plot areas and precisely geolocating plots.

Besides challenges in combining data, methods for scaling tree mortality measures from the individual tree to the scale captured by satellites are also underdeveloped. Whilst freely available remote sensing data provide insights into long‐term forest cover changes (as discussed above), it is hard to relate those trends to trends measured at the plot scale (Fig. 1). High spatial resolution data from various sources (e.g. UAV) can serve as a missing link between tree‐based measures of tree mortality and the broader view offered by spaceborne remote sensing systems (Schiefer *et al*., 2023). However, many high spatial resolution remote sensing data are commercially operated and not freely available to date; and flying targeted airborne or UAV campaigns repeatably over several years is costly and logistically challenging, especially in remote areas where this data would mostly be needed. Those challenges yet limit the usefulness of high spatial resolution data for operationally monitoring tree mortality at large scales. Existing approaches for scaling from trees to satellites are moreover often tailored to specific case studies and lack generalisability. To overcome those existing limitations, a global network of remote sensing super sites (i.e. sites where measurements of tree mortality and ancillary data are made at variable scales simultaneously) might allow for robust and generalisable scaling relationships to be developed. These could, for instance, build on the new GEO‐TREES initiative for assessing biomass (Chave *et al*., 2019; Labrière *et al*., 2023). Finally, remote sensing can also serve as a complementary information stream for enhancing field‐based data analyses of tree mortality trends, such as delivering information on the timing of mortality events between two census dates, on the spatial extent of a mortality event recorded by a plot network, or to target additional ground‐based monitoring.

#### Integrating knowledge

Although monitoring coverage is imperfect, in many regions sufficient data exist to accurately assess the rate of tree mortality. The key is to be able to harness in unison the disparate sources of data available relating to different aspects of tree mortality, forest state, dynamics and health. One step here is to combine measurements of different parts of the system made from different platforms (Beloiu *et al*., 2022). However, true integration of disparate measurement systems is often challenging because of differences in exactly what is being measured (Fig. 1). Process‐based modelling approaches can provide a route to bring together these aspects. Such models are designed to coherently link up equations describing individual processes within forests, based on our best understanding of how they work. The set of processes involved depends on the model, but typically include aspects such as photosynthesis, carbon allocation, growth, competition and disturbances, with resulting rates that differ by type of tree and the environment in which it is located. Constraining the result of one process within these models also imposes a constraint on the rest of the system, allowing information at different scales and on different aspects of the system to be linked together into one coherent picture. Such data integration techniques are increasingly being used with satellite observations for both water and carbon dynamics at various levels of process complexity (Bloom *et al*., 2016; Exbrayat *et al*., 2018; Baatz *et al*., 2021), as well as to initialise tree sizes (Rödig *et al*., 2019). Large‐scale integration of forest inventory observations is less well developed, but some studies exist (Lichstein *et al*., 2014), and the latest vegetation dynamic models with detailed representations of stand structure and forest demography (Smith *et al*., 2014; Argles *et al*., 2020; Koven *et al*., 2020) provide a strong basis for further progress. Method development will be required to solve computational challenges, to appropriately weight different observations in the integration according to spatial representativeness and sampling intensity, and to propagate uncertainty from them (Fer *et al*., 2018; Dokoohaki *et al*., 2022). Relationships from regions constrained by multiple data sources could be applied to better estimate mortality trends in regions where only limited observations (such as optical satellite data) are available. A well‐developed model data integration system could be placed at the centre of a global forest observation system, providing aggregate information on multiple metrics that is analogous to the reanalysis approaches used in meteorology (Hersbach *et al*., 2020).

## A comprehensive and fair global network

Any effort of data integration towards a global assessment of tree mortality will be ultimately limited by spatial and temporal gaps in ground‐based, long‐term forest monitoring. These gaps tend to be larger in low‐income regions of the world and can be largely attributed to the lack of investment in long‐term monitoring and to the challenges of working in remote areas devoid of basic infrastructure, are politically unstable or subject to criminal activities (Nuñez *et al*., 2019; Báldi & Palotás, 2021; Maas *et al*., 2021; Seidler *et al*., 2021). In the tropics, there is also the additional challenge of working in systems of high species diversity, which requires highly qualified professionals in species identification. Different initiatives have tried to fill these gaps by implementing long‐term monitoring sites in tropical regions (e.g. ForestGEO, LTER Brasil, PPBio), as well as integrating and supporting existing local monitoring initiatives (ForestPlots.net *et al*., 2021). Although these efforts have led to invaluable advancement in our understanding of these forests, data gaps remain, and the lack of investment in long‐term monitoring efforts and integration of monitoring into government policies, especially in less wealthy countries, remains a shortcoming.

Forest monitoring in dense, species‐rich and remote tropical systems can be extremely challenging. The identification of species alone can take up to twice the amount of time of recording and measuring the trees. For instance, the establishing of a new monitoring site of 1 ha takes up to 20 person‐days in Central Amazonia, but identifying species, including collecting vouchers by climbers and *ex situ* identification by specialists, can take up to 40 person‐days. Filling spatial gaps may include hiking for up to 5 d or hiring small aircrafts or boats to reach remote regions of continuous forests in Amazonia, the Upper Guinea Forest and the Congo Basin. Being remote can also mean being at risk, not just from potential accidents and diseases but also from potentially violent encounters with poachers, illegal loggers and miners, and armed militia. For these reasons, a whole region with 70 ha of permanent plots was abandoned in 2019 in the English part of Cameroon, of which 58 ha are now in conflict areas and 12 ha have been converted to timber concession. Similarly, *c*. 20 ha of permanent plots in Southern Amazonia cannot be visited since 2018 because of illegal logging and land‐grabbing. This, unfortunately, is not an uncommon situation across tropical regions.

Also problematic is the fact that the capability to locally employ more complex tools like remote sensing (see ‘The role of remote sensing’ section) and process‐based modelling (see the ‘Bringing it all together – data integration across scales’ section) is often limited to wealthier countries. Part of the problem is that the efforts to understand forest functioning across large spatial scales are generally led by scientists whose national context gives greater opportunity to obtain funding for such analyses (Brearley *et al*., 2019; North *et al*., 2020; Asase *et al*., 2022). This creates a power asymmetry in the collaboration between those who collect the data and those who lead the research analysis and papers (Boshoff, 2009). Moreover, this *modus operandi* often discourages scientists from less wealthy countries from sharing the data they collect. One step towards changing this situation is the adoption of co‐design and co‐production practices by those leading the analyses, that is, investing time and resources in discussing plans for analyses and in involving data originators in the analyses, with the necessary capacity building (Mahajan *et al*., 2023).

Global data on forest dynamics will not be comprehensive until the issues around fair scientific collaborations between wealthy and poor countries are acknowledged, addressed and solved. Funding bodies and research institutions unfortunately reinforce and maintain these cultural standards through funding structures and evaluation systems that value individuals over groups, look for fast return on investments and favour short‐term projects. This structure is incompatible with large global collaborations, which are becoming a common way of organising science. A shift in the way global collaborations take place demands large efforts and time commitments that are unlikely to be achieved if they are not appropriately funded (de Lima *et al*., 2022) and evaluated in terms of their collective benefit. An ideal global network should place groups of people at the centre of the collaborative effort and spend a similar (or greater) amount of energy and resources as to what is spent on data analyses on capacity building, particularly of early‐career researchers (Seidler *et al*., 2021). Global initiatives should provide opportunities for all participants to be involved in decision‐making and in the intellectual scientific process. To be truly inclusive, we should revise the current paradigm that focuses on individual scientific leaders, and instead global initiatives should consider adopting a collective mindset mirroring the strategy of science panels. For instance, the IPCC and the Science Panel for the Amazon, which value community effort over that of small teams or individuals, provide models, although it is necessary to ensure that different groups are appropriately represented (Mori, 2022). Governmental efforts, such as the NFIs, have the stability and the long‐term vision needed to provide a platform for the integration of people and data across the globe. Regional efforts to harmonise NFIs across key data gaps are already taking place and similar efforts to integrate NFI and academic communities would be a major step towards closing data gaps.

Different networks have developed strategies to improve fairness in collaboration. For instance, it is now increasingly common to invite all data contributors to participate in the writing processes as authors of manuscripts (e.g. ForestPlots.net) and to list the group as the first author (e.g. ForestPlots.net (ForestPlots.net *et al*., 2021) and DryFlor (DRYFLOR *et al*., 2016)). Although participation in manuscripts is an important step, deeper change will only happen by exchanging knowledge with and transferring resources to less wealthy regions. A fair global network should aim for those in less wealthy regions to lead local to global‐scale analyses and to secure the continuity of field measurements and of their own research agendas. Indeed, a few networks are investing in capacity building by promoting workshops for data contributors (e.g. ForestGEO (Anderson‐Teixeira *et al*., 2015); International Long Term Ecological Research, ILTER). It is also key to have the mode of collaboration and data sharing well defined, with roles written and agreed by all members (sPlots (Bruelheide *et al*., 2019), ForestPlots.net (ForestPlots.net *et al*., 2021)). These first steps are extremely important, but they are only the beginning if we are to advance global science in an equitable manner. The steps towards fair, truly inclusive collaborations need to be encouraged and recognised by the scientific community and funding agencies. Only then, will we be able to achieve a comprehensive global understanding of tree mortality trends.

## Vision – what we need to do as a community

**Fig. 4:**
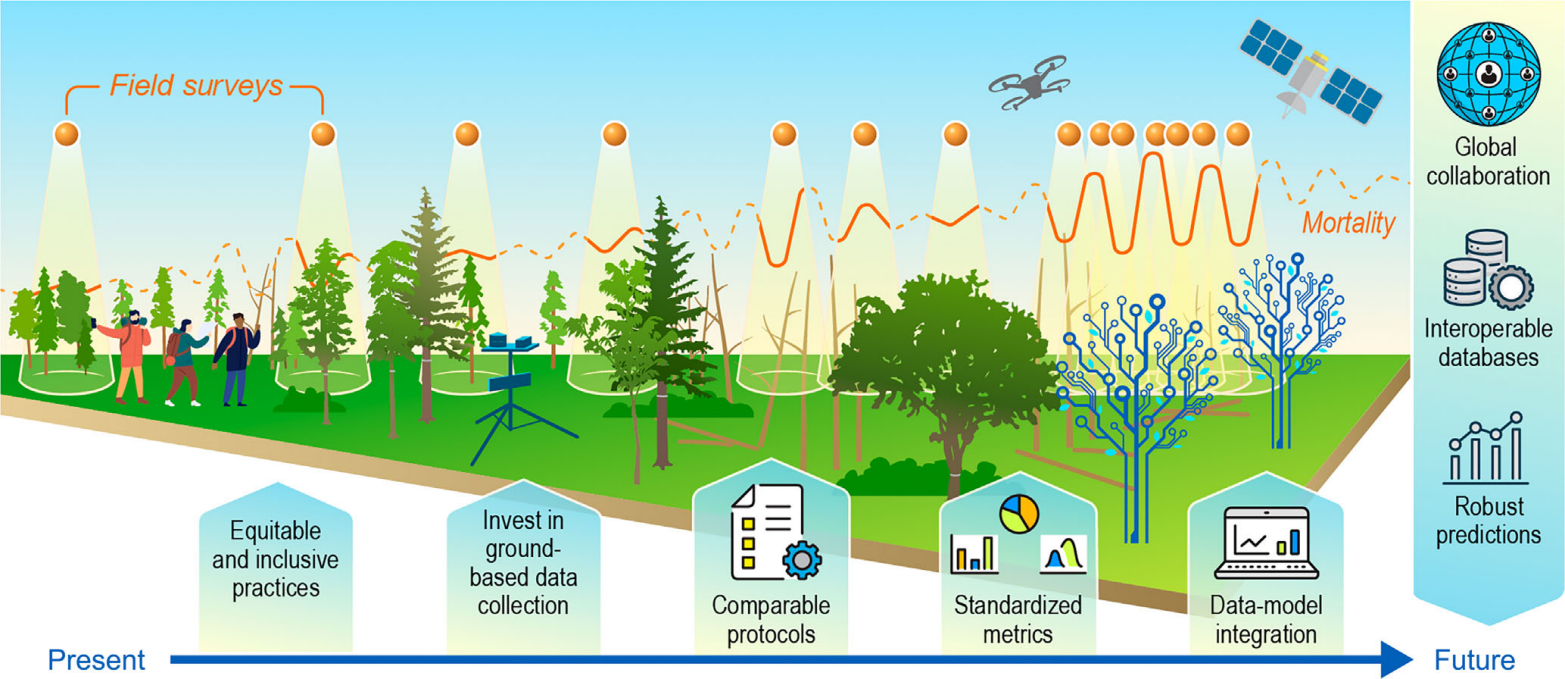
Roadmap towards a global understanding of tree mortality. The five major steps integrate long‐term field data with remote sensing and modelling techniques to build a fair, diverse and equitable system to monitor tree mortality at global scales. At its core, this system is built in a progressive fashion that over time (left to right in the figure) allows for expanding current monitoring frameworks, both spatially and temporarily, to enhance scientific and social collaborations via interoperable databases that ultimately will help develop stronger predictions on tree mortality, its trends and main drivers.

A global monitoring system of tree mortality requires the harmonisation of existing global long‐term field data and their integration with remote sensing and modelling techniques to gap‐fill these data across time and space (Fig. 4). This requires development of methods and agreements enabling seamless flows of information from the field to global assessments. Whilst there is a wealth of established plots that could form the backbone of a global tree mortality monitoring system if funding continues (Fig. 3), some data networks might require adjustments to their protocols to substantially improve monitoring of tree death. This includes increasing the temporal resolution of data collection and shifting towards protocols that track individual trees and characterise the condition of both live and dead trees (i.e. standing, falling, uprooted or logged). The spatial and temporal gaps in forest inventory plots remain a major limitation to an operational tree mortality monitoring system. Not only hypothesis‐driven research, but also data collection, needs to be a priority with funding bodies to better support the implementation and continuity of long‐term ecological monitoring programmes (e.g. Programa de Pesquisas Ecológicas de Longa Duração, PELD). Remote sensing techniques should be used to detect areas where forest canopy is changing, helping to target future ground‐based work and fill in temporal and spatial gaps.

As a global effort, this must be used as an opportunity to advance towards an equitable scientific community. Funding agencies must invest in forest monitoring in data‐gap areas, mostly located in less wealthy countries, whilst promoting fair collaborations and capacity building that empower local scientists. The format of science panels (i.e. the IPCC and IPBES) should allow more inclusive practices when compared to research papers led by a few individuals and for results to feed quickly into policy making. We call for a global tree mortality monitoring system to be supported by multilateral organisations, such as the UN and the FAO, providing for the long‐term maintenance of this global effort. Our current understanding of forests, the advancement of new technologies and world‐wide connectivity means that now a global monitoring system of tree mortality is not just urgently needed but also feasible. In summary, we propose the following roadmap towards a global understanding of tree mortality, building on our minimum requirements (Fig. 4):

1. Promote equitable practices across the community that empower those collecting the data.
2. Invest in ground‐based data collection, sustaining long‐term efforts and expanding to data‐poor regions.
3. Adjust protocols to facilitate comparability and improve quantification of rates and causes of tree mortality.
4. Generate standardised tree mortality metrics from ground‐based data that can be widely used by the scientific community and facilitate comparability across studies.
5. Integrate ground‐based data with remote sensing data and process‐based models to expand current observations temporally and spatially and understand their underlying drivers.

Following this roadmap will allow us to create interoperable datasets on tree mortality globally through fair collaboration and ultimately lead to robust predictions of tree mortality trends.

## Competing interests

None declared.

## Author contributions

CS, AE‐M and TAMP initiated the publication with help of BS, HH, NR and RS. CS, AE‐M and TAMP wrote the manuscript. WRLA, KJA‐T, GA, MBS, BJB, HJB, BB‐L, KMB, FQB, FB, MC, JJC, GC, FRC, RD, HD, SJD, SD, BPD, RAFdL, MF, JBF, MG, ALdG, AG, GSG, JLG, FMPG, LG, AGG, EGC, WMH, HH, MLH, AH, JH, MJH, C‐yH, BH, T Jackson, T Jucker, ASJ, SJ, TK, JK, MMK, KK, NLP, L‐TL, RLC, EEM, JMD, EHM, JM‐V, NM, PWM, ASM, MAM, Jan‐Peter Mund, RM, MM‐T, SCM, TAN, SN, CAN, MNS, MJO, JP, GLWP, OLP, JMP, RRR, AR, GXR, NKR, PR‐B, KXR, CS‐E, TGM Sanders, RSB, T Scharnweber, M‐JS, BS, S Schwarz, RS, ES, ACS, GS, J Socha, KS, J Stillhard, DBS, S Suvanto, MS, MS‐P, AJT, ART, FTF, GV, ACV, AV, EV, LTW, SKW, KY, MAZ, LZO, DZ, ABdC‐F, EvdM and MvdM contributed to several workshops discussing the individual subsections of the manuscript and they all revised the manuscript.

## Disclaimer

The New Phytologist Foundation remains neutral with regard to jurisdictional claims in maps and in any institutional affiliations.

## Data Availability

All data and code are available under: https://zenodo.org/records/13221241.

## Appendix A1

### International Tree Mortality Network

Cornelius Senf (0000‐0002‐2389‐2158)^1^, Adriane Esquivel‐Muelbert (0000‐0001‐5335‐1259)^2,3^, Thomas A. M. Pugh (0000‐0002‐6242‐7371)^2,3,4^, William R. L. Anderegg (0000‐0001‐6551‐3331)^5,6^, Kristina J. Anderson‐Teixeira (0000‐0001‐8461‐9713)^7,8^, Gabriel Arellano (0000‐0003‐3990‐5344)^9,10^, Mirela Beloiu Schwenke (0000‐0002‐3592‐8170)^11^, Barbara J. Bentz (0000‐0002‐2741‐1542)^12^, Hans Juergen Boehmer (0000‐0002‐9176‐4836)^13,14^, Ben Bond‐Lamberty (0000‐0001‐9525‐4633)^15^ Kauane Maiara Bordin (0000‐0003‐3871‐6293)^16^, Francis Q. Brearley^17^, Filippo Bussotti (0000‐0002‐8353‐4459)^18^, Maxime Cailleret (0000‐0001‐6561‐1943)^19^, J. Julio Camarero (0000‐0003‐2436‐2922)^20^, Gherardo Chirici (0000‐0002‐0669‐5726)^18,21^, Flavia R. C. Costa^22^, Ricardo Dalagnol (0000‐0002‐7151‐8697)^23,24^, Hendrik Davi (0000‐0001‐8828‐3145)^25^, Stuart J. Davies (0000‐0002‐8596‐7522)^8^, Sylvain Delzon (0000‐0003‐3442‐1711)^26^, Bishnu Prasad Dhakal (0000‐0002‐8534‐6558)^27^, Renato A. Ferreira de Lima (0000‐0002‐1048‐0138)^28^, Marco Ferretti (0000‐0002‐8488‐0804)^29^, Joseph B. Fontaine (0000‐0002‐6515‐7864)^30^, Matteo Garbarino (0000‐0002‐9010‐1731)^31^, André Luís de Gasper (0000‐0002‐1940‐9581)^32^, Arthur Gessler (0000‐0002‐1910‐9589)^33,34^, Gregory S. Gilbert (0000‐0002‐5195‐9903)^35^, John L. Godlee (0000‐0001‐5595‐255X)^36^, Francisco Maiato Pedro Gonçalves^37,38^, Leen Govaere^39^, Alvaro G. Gutiérrez (0000‐0001‐8928‐3198)^40,41^, Ernesto Gómez Cardozo (0000‐0002‐6846‐4106)^42^, William M. Hammond (0000‐0002‐2904‐810X)^43^, Henrik Hartmann (0000‐0002‐9926‐5484)^44,45^, Martina L. Hobi (0000‐0003‐3537‐9738)^46^, Andrés Holz (0000‐0002‐8587‐2603)^47^, Jürgen Homeier (0000‐0001‐5676‐3267)^48,49^, Mark Joseph Hovenden (0000‐0001‐7208‐9700)^50^, Cho‐ying Huang (0000‐0002‐9174‐7542)^51,52^, Bruno Hérault (0000‐0002‐6950‐7286)^53,54^, Toby Jackson (0000‐0001‐8143‐6161)^55^, Tommaso Jucker (0000‐0002‐0751‐6312)^55^, Alistair S. Jump (0000‐0002‐2167‐6451)^56^, Samuli Junttila (0000‐0001‐8276‐9259)^57^, Teja Kattenborn (0000‐0001‐7381‐3828)^58^, Joice Klipel (0000‐0003‐3936‐9692)^16,59^, Martyna M. Kotowska (0000‐0002‐2283‐5979)^49,60^, Kamil Král (0000‐0002‐3848‐2119)^61^, Nicola La Porta (0000‐0002‐7080‐3349)^62,63^, Leonel Lopez‐Toledo (0000‐0003‐3424‐5746)^64^, René López‐Camacho (0000‐0003‐2026‐0371)^65^, Eduardo Eiji Maeda (0000‐0001‐7932‐1824)^66,67^, Jesús Mallol Díaz^68^, Emanuel H. Martin (0000‐0003‐0432‐6262)^69^, Jordi Martínez‐Vilalta (0000‐0002‐2332‐7298)^70,71^, Nate McDowell^72,73^, Peter W. Moonlight (0000‐0003‐4342‐2089)^74,75^, Akira S. Mori (0000‐0002‐8422‐1198)^76^, Mohd Afzanizam Muda (0000‐0003‐1374‐2305)^77^, Jan‐Peter Mund (0000‐0002‐4878‐5519)^78^, Robert Muscarella (0000‐0003‐3039‐1076)^79^, Moisés Méndez‐Toribio (0000‐0002‐8568‐8072)^80,81^, Sandra C. Müller (0000‐0002‐6316‐2897)^82^, Thomas A. Nagel (0000‐0002‐4207‐9218)^83^, Stefan Neagu (0000‐0002‐2399‐3864)^84,85^, Charles Andrew Nock (0000‐0002‐3483‐0390)^86^, Moses Nsanyi Sainge (0000‐0003‐1677‐3043)^87^, Michael J. O'Brien^88^, Josep Peñuelas (0000‐0002‐7215‐0150)^70,89^, George L. W. Perry (0000‐0001‐9672‐9135)^90^, Oliver L. Phillips (0000‐0002‐8993‐6168)^91^, Juan Manuel Posada^92^, Ricardo Ribeiro Rodrigues (0000‐0003‐4818‐0736)^93^, Anamaria Roman (0000‐0001‐9674‐6461)^94^, Guillaume Xavier Rousseau^42,95^, Nadine Katrin Ruehr (0000‐0001‐5989‐7463)^96,97^, Paloma Ruiz‐Benito (0000‐0002‐2781‐5870)^98,99^, Katinka X. Ruthrof (0000‐0003‐2038‐2264)^30,100^, Christian Salas‐Eljatib (0000‐0002‐8468‐0829)^101,102^, Tanja G. M. Sanders (0000‐0002‐4536‐4540)^103^, Rodrigo Scarton Bergamin (0000‐0002‐2405‐9977)^2,104^, Tobias Scharnweber (0000‐0002‐4933‐5296)^105,‡^, Mart‐Jan Schelhaas (0000‐0003‐4525‐2677)^106^, Bernhard Schuldt (0000‐0003‐4738‐5289)^107^, Selina Schwarz (0000‐0001‐6042‐1649)^108^, Rupert Seidl (0000‐0002‐3338‐3402)^109,110^, Ekaterina Shorohova (0000‐0002‐8238‐927X)^111^, Ana Carolina Silva^112^, Geert Sioen (0000‐0001‐5590‐3512)^113^, Jarosław Socha (0000‐0002‐9568‐5764)^114^, Krzysztof Stereńczak (0000‐0002‐9556‐0144)^115^, Jonas Stillhard (0000‐0001‐8850‐4817)^29^, Dejan B. Stojanović^116^, Susanne Suvanto (0000‐0002‐0345‐3596)^2,104,111^, Miroslav Svoboda (0000‐0003‐4050‐3422)^117^, Martina Sánchez‐Pinillos (0000‐0002‐1499‐4507)^118,119^, Andrew J. Tanentzap^120^, Anthony R. Taylor (000‐0002‐2122‐6792)^121^, Fabiano Turini Farah (0000‐0003‐2406‐8766)^122^, Giorgio Vacchiano (0000‐0001‐8100‐0659)^123^, Alexander C. Vibrans (0000‐0002‐8789‐5833)^124^, Alberto Vilagrosa (0000‐0002‐1432‐1214)^125,126^, Emilio Vilanova (0000‐0001‐6289‐5127)^127^, Lars T. Waser (0000‐0002‐2609‐9147)^29^, Susan K. Wiser (0000‐0002‐8938‐8181)^128^, Kailiang Yu^129^, Miguel A. Zavala (0000‐0003‐1456‐0132)^98^, Laio Zimermann Oliveira^130^, Daniel Zuleta (0000‐0001‐9832‐6188)^131,132^, Alvaro Boson de Castro‐Faria (0000‐0001‐6276‐0898)^133^, Ernst van der Maaten (0000‐0002‐5218‐6682)^134^, Marieke van der Maaten‐Theunissen (0000‐0002‐2942‐9180)^134^

1. Technical University of Munich, School of Life Sciences, Earth Observation for Ecosystem Management, Hans‐Carl‐von‐Carlowitz‐Platz 2, Freising, 85354, Germany
2. School of Geography, Earth and Environmental Sciences, University of Birmingham, Birmingham, B15 2TT, UK
3. Birmingham Institute of Forest Research (BIFoR), University of Birmingham, Birmingham, B15 2TT, UK
4. Department of Physical Geography and Ecosystem Science, Lund University, Box 117, Lund, 22100, Sweden
5. Wilkes Center for Climate Science and Policy, University of Utah, 1390 Presidents Circle, Salt Lake City, UT, 84103, USA
6. School of Biological Sciences, University of Utah, 257 South 1400 East, Salt Lake City, UT, 84103, USA
7. Conservation Ecology Center, Smithsonian's National Zoo & Conservation Biology Institute, Front Royal, VA, 22630, USA
8. ForestGEO, Smithsonian Tropical Research Institute, West Loading Dock MRC‐166, 10th and Constitution Ave, Washington, DC, 20560, USA
9. Ecology and Evolutionary Biology, University of Michigan, 1105 N University Ave, Ann Arbor, MI, 48109, USA
10. Oikobit LLC, 2105 Vista Oeste St NW, Albuquerque, NM, 87120, USA
11. Department of Environmental Systems Science, ETH Zurich, Universitätstrasse 16, Zürich, 8092, Switzerland
12. USDA Forest Service, Rocky Mountain Research Station, 860 N 1200 E, Logan, UT, 84321, USA
13. Institute of Earth System Sciences – Geobotany Section, Leibniz University Hannover, Nienburger Straße 17, Hannover, 30167, Germany
14. School of Agriculture, Geography, Environment, Ocean and Natural Sciences, The University of the South Pacific, Laucala Bay, Suva, Fiji
15. Joint Global Change Research Institute, Pacific Northwest National Laboratory, 5825 University Research Ct. #3500, College Park, MD, 20740, USA
16. Plant Ecology Lab, Ecology Department, Universidade Federal do Rio Grande do Sul, Bento Gonçalves Ave, 9500, Porto Alegre, 90650‐001, Brazil
17. Department of Natural Sciences, Manchester Metropolitan University, Chester Str., Manchester, M1 5GD, UK
18. Dipartimento di Scienze e Tecnologie Agrarie, Alimentari, Ambientali e Forestali, Università degli Studi di Firenze, Piazzale delle Cascine 18, Florence, 50144, Italy
19. Aix Marseille Univ, INRAE, UMR RECOVER, 3275 route de Cézanne, CS 40061, Aix‐en‐Provence, Cedex 5, France
20. Instituto Pirenaico de Ecología (IPE‐CSIC), Avda. Montañana 1005, Zaragoza, 50059, Spain
21. Fondazione per il Futuro delle Città, Via di Novoli, 10, Florence, 50127, Italy
22. Instituto Nacional de Pesquisas da Amazônia, Coordenação de Pesquisas em Dinâmica Ambiental, Av André Araújo, 2936, Manaus, CEP 69067‐375, Brazil
23. CTrees, 12S Raymond Ave, Pasadena, CA, 91105, USA
24. NASA‐Jet Propulsion Laboratory, California Institute of Technology, Pasadena, CA, 91109, USA
25. URFM, Ecologie des Forêts méditerranéennes, UR629, Agroparc, CS 40509, 84914, Avignon, Cedex 9, France
26. University of Bordeaux, INRAE, UMR BIOGECO, Pessac, 33615, France
27. Forest Research and Training Centre, Babarmahal, Kathmandu, 44600, Nepal
28. Departamento de Ciências Biológicas, ESALQ, Universidade de São Paulo, Avenida Pádua Dias, 11, Piracicaba, 13418‐900, Brazil
29. Swiss Federal Institute for Forest, Snow and Landscape Research WSL, Zuercherstrasse 111, Birmensdorf, 8903, Switzerland
30. School of Environmental and Conservation Sciences, Murdoch University, 90 South St, Murdoch, WA, 6150, Australia
31. DISAFA Department, University of Torino, Largo P. Braccini 2, Grugliasco, TO, 10095, Italy
32. Departamento de Ciências Naturais, Universidade Regional de Blumenau, Blumenau, Santa Catarina, 89030‐903, Brazil
33. Institute of Terrestrial Ecosystems, ETH Zurich, Universitätstrasse 22, Zurich, 8092, Switzerland
34. Forest Dynamics, Swiss Federal Research Institute WSL, Zürcherstrasse 111, Birmensdorf, CH‐8903, Switzerland
35. University of California, Santa Cruz, 1156 High St, Santa Cruz, CA, 95064, USA
36. School of GeoSciences, University of Edinburgh, Edinburgh, EH9 3FF, UK
37. Universidade Mandume Ya Ndemufayo, Av. Hoji Ya Henda, No. 30, Lubango, Angola
38. Herbário do Lubango, ISCED‐Huíla, Rua Sarmento Rodrigues S/N, Lubango, Angola
39. Agency for Nature and Forests, Havenlaan 88 bus 75, Brussel, 1000, Belgium
40. Departamento de Ciencias Ambientales y Recursos Naturales Renovables, Facultad de Ciencias Agronómicas, Universidad de Chile, Av. Santa Rosa 11315, La Pintana, Santiago, 8820808, Chile
41. Institute of Ecology and Biodiversity (IEB), Avenue Libertador Bernardo O´Higgins 340, Santiago, 8320165, Chile
42. Postgraduate Program in Agroecology, Maranhão State University (UEMA), Av. Lourenço Vieira da Silva 1000, Jardim São Cristovão, São Luís, MA, 65055‐310, Brazil
43. Agronomy Department, Institute of Food and Agricultural Sciences, 1676 McCarty Drice, Gainesville, FL, 32611, USA
44. Institute for Forest Protection, Julius Kuehn‐Institute – Federal Research Centre for Cultivated Plants, Erwin‐Baur Str. 27, Quedlinburg, 06484, Germany
45. Department of Biogeochemical Processes, Max Planck Institute for Biogeochemistry, Hans‐Knoell‐Str. 10, Jena, 07743, Germany
46. Forest Resources and Management, Swiss Federal Institute for Forest, Snow and Landscape Research WSL, Birmensdorf, 8903, Switzerland
47. School of the Earth, Environment, and Society (Geography), 1721 SW Broadway, Portland, OR, 97201, USA
48. Faculty of Resource Management, HAWK University of Applied Sciences and Arts, Göttingen, 37077, Germany
49. Department of Plant Ecology and Ecosystems Research, University of Goettingen, Untere Karspüle 2, Göttingen, 37073, Germany
50. Biological Sciences, School of Natural Sciences, University of Tasmania, Locked Bag 55, Hobart, Tas., 7001, Australia
51. Department of Geography, National Taiwan University, 1 Sec. 4 Roosevelt Road, Taipei, 10617, Taiwan
52. Research Center for Future Earth, National Taiwan University, 1 Sec. 4 Roosevelt Road, Taipei, 10617, Taiwan
53. CIRAD, UPR Forêts et Sociétés, Montpellier, F‐34398, France
54. Forêts et Sociétés, Univ Montpellier, CIRAD, Montpellier, F‐34398, France
55. School of Biological Sciences, University of Bristol, Bristol, BS8 1TQ, UK
56. Biological and Environmental Sciences, Faculty of Natural Sciences, University of Stirling, Stirling, FK9 4LA, UK
57. School of Forest Sciences, University of Eastern Finland, Yliopistokatu 2, Joensuu, 80101, Finland
58. Sensor‐based Geoinformatics (geosense), University of Freiburg, Tennenbacherstr. 4, Freiburg, 79106, Germany
59. Leuphana University of Lüneburg, Institute of Ecology, Universitätsallee 1, Lüneburg, 21335, Germany
60. School of Natural Sciences, Macquarie University, North Ryde, NSW, 2109, Australia
61. Department of Forest Ecology, Silva Tarouca Research Institute, Lidická 25/27, Brno, 60200, Czech Republic
62. Research and Innovation Centre, Fondazione Edmund Mach (FEM), Via Mach 1, Trento, San Michele all'Adige, 38010, Italy
63. MOUNTFOR Project Centre, European Forest Institute, Via E. Mach 1, Trento, San Michele all'Adige, 38010, Italy
64. Universidad Michoacana de San Nicolas de Hidalgo, Av. San Juanito Itzicuaro s/n, Morelia, 58195, Mexico
65. Universidad Distrital Francisco José de Caldas, Carrera 5 Este N° 15‐82, Bogotá, 4000, Colombia
66. Department of Geosciences and Geography, University of Helsinki, Gustaf Hällströmin katu 2, Helsinki, 00014, Finland
67. Finnish Meteorological Institute, FMI, P.O. Box 503, Helsinki, FI‐00101, Finland
68. Department of Physical Geography and Ecosystem Science, Lund University, Lund, 22362, Sweden
69. College of African Wildlife Management, Mweka, P. O. Box, Moshi, 3031, Tanzania
70. CREAF, Cerdanyola del Vallès, Barcelona, Catalonia, 08193, Spain
71. Universitat Autònoma de Barcelona, Bellaterra (Cerdanyola del Vallès), Catalonia, E08193, Spain
72. Atmospheric Sciences and Global Change Division, Pacific Northwest National Lab, PO Box 999, Richland, WA, 99352, USA
73. School of Biological Sciences, Washington State University, PO Box 644236, Pullman, WA, 99164‐4236, USA
74. Botany, School of Natural Sciences, Trinity College Dublin, Dublin 2, Ireland
75. Royal Botanic Garden Edinburgh, 20A Inverleith Row, Edinburgh, EH5 3RL, UK
76. Research Center for Advanced Science and Technology, University of Tokyo, 4‐6‐1 Komaba, Meguro, Tokyo, 153‐8904, Japan
77. Forestry Department of Peninsular Malaysia (JPSM), State Project Management Office (SPMO) Selangor SMPEM Project, Pejabat Pembangunan Dan Latihan Perhutanan Sungai Buloh, KM21, Jalan Subang, Sungai Buloh, Selangor, 47000, Malaysia
78. Eberswalde University for Sustainable Development, Schicklerstrasse 5, Eberswalde, 16225, Germany
79. Department of Ecology and Genetics, Uppsala University, Kåbovägen 4 house 7, Uppsala, 75236, Sweden
80. Instituto de Ecología, A.C.‐Centro Regional del Bajío, Red de Diversidad Biológica del Occidente Mexicano, Pátzcuaro, 61600, Mexico
81. Consejo Nacional de Humanidades, Ciencias y Tecnologías (CONAHCYT), Mexico City, 03940, Mexico
82. Departamento de Ecologia, Instituto de Biociências, Universidade Federal do Rio Grande do Sul, Av. Bento Gonçalves, 9500, Porto Alegre, 90650‐001, Brazil
83. Department of forestry and renewable forest resources, Biotechnical Faculty, University of Ljubljana, Jamnikarjeva 101, Ljubljana, 1000, Slovenia
84. National Forestry Research‐Development Institute, Bd. Eroilor 128, Voluntari, 077190, Romania
85. University of Agronomic Sciences and Veterinary Medicine of Bucharest, 59 Mărăşti Boulevard, Bucharest, 011464, Romania
86. Department of Renewable Resources, College of Natural and Applied Sciences, University of Alberta, 116 St and 85 Ave, Edmonton, AB, T6G 2R3, Canada
87. Reptile and Amphibian Program Sierra Leone (RAP‐SL), 7 McCauley Str. Murray Town, Freetown, Sierra Leone
88. Estación Experimental de Zonas Áridas, Consejo Superior de Investigaciones Científicas, Carretera de Sacramento s/n, La Cañada, Almería, 04120, Spain
89. CSIC, Global Ecology Unit CREAF‐CSIC‐UAB, Bellaterra, Barcelona, Catalonia, 08193, Spain
90. School of Environment, Private Bag 92019, Auckland, 1142, New Zealand
91. School of Geography, University of Leeds, Leeds, LS2 9JT, UK
92. Biology Department, Faculty of Natural Sciences, Universidad del Rosario, Carrera 24 No. 63C‐69, Bogotá, 111221, Colombia
93. Universidade de São Paulo, Escola Superior de Agricultura Luiz de Queiroz, Laboratório de Ecologia e Restauração Florestal, Av. Pádua Dias n 11, LCB, Piracicaba, 13418‐900, Brazil
94. Institute of Biological Research Cluj, Branch of NIRDBS, 48 Republicii St., Cluj‐Napoca 400015, Romania
95. Soil Biology Laboratory (LABS), UEMA, Av. Lourenço Vieira da Silva 1000, Jardim São Cristovão, São Luís, MA, 65055‐310, Brazil
96. Karlsruhe Institute of Technology, KIT‐Campus Alpin, Kreuzeckbahnstrasse 19, Garmisch‐Partenkirchen, 82467, Germany
97. Karlsruhe Institute of Technology, Institute of Geography and Geoecology, Kaiserstrasse 12, Garmisch‐Partenkirchen, 82467, Germany
98. Departamento de Ciencias de la Vida, Universidad de Alcalá, Grupo de Ecología y Restauración Forestal (FORECO), Alcalá de Henares, Madrid, 28805, Spain
99. Departamento de Geología, Geografía y Medio Ambiente, Universidad de Alcalá, Grupo de Investigación en Teledetección Ambiental, Alcalá de Henares, Madrid, 28801, Spain
100. Department of Biodiversity, Conservation and Attractions, Biodiversity and Conservation Science, 17 Dick Perry Ave, Kensington, WA, 6151, Australia
101. Departamento de Gestión Forestal y su Medio Ambiente, Universidad de Chile, Santiago, 8330015, Chile
102. Vicerrectoría de Investigación y Postgrado, Universidad de La Frontera, Avenida Francisco Salazar, Temuco, 01145, Chile
103. Thünen‐Institute of Forest Ecosystems, Alfred‐Möller‐Str. 1, Eberswalde, 16225, Germany
104. Birmingham Institute of Forest Research (BIFoR), University of Birmigham, Birmigham, B15 2TT, UK
105. Institute of Botany and Landscape Ecology, University of Greifswald, Soldmannstr.15, Greifswald, 17489, Germany
106. Wageningen Environmental Research (WENR), Wageningen University and Research, Droevendaalsesteeg 3, Wageningen, 6708PB, The Netherlands
107. Institute of Forest Botany and Forest Zoology, Chair of Forest Botany, Technical University of Dresden, Pienner Str. 7, Tharandt, 01737, Germany
108. Karlsruhe Institute of Technology (KIT), Institute of Meteorology and Climate Research – Atmospheric Environmental Research (IMK‐IFU), Kreuzeckbahnstraße 19, Garmisch‐Partenkirchen, 82467, Germany
109. Technical University of Munich, School of Life Sciences, Ecosystem Dynamics and Forest Management Group, Hans‐Carl‐von‐Carlowitz‐Platz 2, Freising, 85354, Germany
110. Berchtesgaden National Park, Doktorberg 6, Berchtesgaden, 83471, Germany
111. Natural Resources Institute Finland (Luke), Latokartanonkaari 9, Helsinki, 00790, Finland
112. Universidade do Estado de Santa Catarina, Av. Luiz de Camões 2090, Lages, 88520‐000, Brazil
113. Research Institute for Nature and Forest (INBO), Havenlaan 88/73, Brussels, 1000, Belgium
114. University of Agriculture in Krakow, Al. Mickiewicza 21, Krakow, 31‐120, Poland
115. Department of Geomatics, Forest Research Institute, Braci Leśnej 3 St., Sękocin Stary, Raszyn, 05‐090, Poland
116. Institute of Lowland Forestry and Environment, University of Novi Sad, Antona Cehova 13d, Novi Sad, 21102, Serbia
117. Faculty of Forestry and Wood Sciences, Czech University of Life Sciences, Kamycka 129, Praha 6, Suchdol, 16521, Czech Republic
118. ISEM CNRS Univ. Montpellier, Pl. E. Bataillon, Montpellier, 34090, France
119. CEF UQAM, Av. Président Kénnedy 141, Montreal, QC, H2X 1Y4, Canada
120. Ecosystems and Global Change Group, School of the Environment, Trent University, 1600 West Bank Dr., Peterborough, ON, K9L 0G2, Canada
121. Faculty of Forestry and Environmental Management, University of New Brunswick, 28 Dineen Dr., Fredericton, E3B 5A3, Canada
122. Re.green, Juliano Bellini Road, Piracicaba, 13427‐226, Brazil
123. DISAA ‐ Università di Milano, via Celoria 2, Milan, 20133, Italy
124. Department of Forest Engineering, Universidade Regional de Blumenau, Rua São Paulo 3250, Blumenau, 89030‐000, Brazil
125. Mediterranean Center for Environmental Studies (CEAM Foundation), Joint Research Unit University of Alicante‐CEAM, University of Alicante, Sant Vicent del Raspeig, Alicante, 03690, Spain
126. Department of Ecology, University of Alicante, Sant Vicent del Raspeig, Alicante, 03690, Spain
127. Wildlife Conservation Society (WCS), 2300 Southern Boulevard, Bronx, NY, 10460, USA
128. Manaaki Whenua – Landcare Research, 74 Gerald St., Lincoln, 7608, New Zealand
129. High Meadows Environmental Institute, Princeton University, Princeton, NJ, 08544, USA
130. Universidade Regional de Blumenau, Rua São Paulo 3250, Blumenau, 89030‐000, Brazil
131. Department of Biological and Environmental Sciences, University of Gothenburg, Gothenburg, Box 100, Gothenburg, 40530, Sweden
132. Forest Global Earth Observatory, Smithsonian Tropical Research Institute, West Loading Dock MRC‐166, 10^th^ and Constitution Ave, NW Washington, DC, 20560, USA
133. Universidade Tecnológica Federal do Paraná (UTFPR), Boa‐Esperança Road, 4^th^ Kilometer, Municipality of Dois Vizinhos, State of Paraná, 85660‐000, Brazil
134. Chair of Forest Growth and Woody Biomass Production, TU Dresden, Dresden, 01062, Germany
